# Healthcare professionals’ views of the experiences of individuals living with Crohn’s Disease in Spain. A qualitative study

**DOI:** 10.1371/journal.pone.0190980

**Published:** 2018-01-23

**Authors:** Sofía García-Sanjuán, Manuel Lillo-Crespo, Miguel Richart-Martínez, Ángela Sanjuán-Quiles

**Affiliations:** Nursing Department, Health Sciences Faculty, University of Alicante, San Vicente del Raspeig, Spain; University Hospital Llandough, UNITED KINGDOM

## Abstract

Crohn’s Disease (CD) in Spain lacks of a unified National Clinical Pathway and not even any early detection program and professional follow-up outpatient attention once it has been diagnosed. Little is known about the Spanish health professionals’ views of the experiences of individuals living with Crohn’s Disease nationwide and also about how the Spanish Health System faces this situation. A qualitative research method was conducted to explore this topic through in-depth interviews with eleven healthcare professionals, who represented different clinics treating people with CD from the province of Alicante (Spain). Three topics and seven sub-topics were derived from the analysis of the content emerging from the interviews. The three main topics were: the healthcare system as a hindrance for ongoing treatment of CD, the impact of the disease, support networks. The knowledge of CD gained by healthcare professionals, in the contexts studied here within, with regards to the psychosocial aspects and the experience of those living with the disease and their immediate circles, is poor, if not null on an academic level, becoming experiential on their incorporation into the professional field. Additionally, a priori, they lack the tools to address the doubts and concerns of patients from the moment of diagnosis through the ongoing care of the patient. Organizational hindrances, such as the lack of time and consensual guidelines for adequately monitoring CD patients in Alicante (Spain), further restrict the patient-professional relationship. Due to the consensus established by the National Agency regulating the contents of the Health Professions’ Education and Training across the country, we are assuming that the phenomenon highlighted may be similar in other parts of Spain. Therefore, it can be said that healthcare professionals have a limited understanding of the impact of CD on the day-to-day life of those affected, not being considered a part of the CD patients’ formal support network. Nonetheless, they are conscious of this limitation and advocate for multidisciplinary teams as the best means of attending to people living with CD. Our study outcomes may represent the first step onto identifying strategies and best practices for establishing an effective therapeutic relationship, as well as any hindering factors.

## Introduction

Crohn´s Disease (CD) falls within the category of inflammatory bowel disease (IBD), known for comprising a group of clinical signs and symptoms characterized by chronic inflammatory processes of unknown aetiology [1]. The annual incidence of CD is 12.7 per 100,000 people-year in Europe, 5.0 per 100,000 people-year in Asia and the Middle East and 20.2 per 100,000 people-year in North America [2]. Its prevalence in Europe is still on the rise, with one million people currently estimated to be suffering from CD [3].

Crohn´s Disease is characterized by a focal, granulomatous inflammation of the digestive tract, which generally progresses, more or less rapidly, from a chronic form towards stenosing fibrosis, abscesses and fistulas [4]. It often arises in adolescence or early adulthood, from 15–35 years of age, with no gender differences. It poses an important public health issue given its frequency, chronic character and potential severity [3,5]. Despite the risk of fatality being low, at times quality of life is severely altered for CD patients who must constantly struggle with the disease, faced with an uncertain future and reduced levels of energy, which in turn restricts them from partaking in activities they enjoyed prior to developing the disease and the expectation in most cases of eventually requiring a colostomy bag [6,7]. The management and treatment of this disease requires an integrated approach, effective coordination among professional care providers and the education of the patient and their immediate circles [8]. Such needs do not appear to be met, both in the patients’ own words [9–13] and those of the healthcare professionals who, despite having international references such as the clinical guides NICE and ECCO [14–16], do not have a common clinical pathway, useful for a range of contexts, at their disposal across all healthcare departments on a national level. Despite efforts being made in Spain to achieve an effective data communication system, due to a lack of coordination between healthcare professionals from primary healthcare centres and hospitals, those affected by CD are often left unsupported by the General Healthcare Law of 1986 (Ley General de Sanidad de 1986) in which it was determined that all Spanish citizens have the right to 24-hour-a-day coordinated healthcare assistance [17,18]. Therefore, the aim of this paper is to explore health care professionals’ views of the experiences of individuals living with Crohn’s Disease in Spain.

## Materials and methods

A qualitative descriptive study was utilized [19] which involved individual interviews conducted on healthcare professionals directly participating in the provision of care to people with CD. Data was generated via in-depth interviews performed by an experienced researcher, which were recorded, transcribed textually and analyzed by three independent researchers who did not participate in the interviews, thus producing a triangulated data analysis.

### Ethical declaration

This study was approved by the Ethics Committee of the University of Alicante (Reference number: UA-2016-06-20). All participants were informed by the researcher of the aim of the study, the methods used and how they would participate. Prior to being interviewed, informed consent was obtained in writing from those participating, as well as the acceptance from the institutions to which the personnel interviewed belonged to.

### Sample

The study sample were healthcare professionals who work with people affected by CD from the province of Alicante, (Spain) whether from public or private institutions. The sample was selected from medical and nursing staff via a non-probabilistic intentional sampling method including professionals that were involved in the CD patient journey and achieving the following inclusion criteria: frequent contact and a minimum 6 months experience with CD patients. Finally 11 healthcare professionals were selected to participate in the study. The defining characteristics of these participants are shown in Table 1.

**Table 1:** Characteristics of the participants.

Healthcare professionals
3 Specialist doctor in digestive system 1 Surgeron 1 Primary/community doctor 1 Primary/community nurse 1 Secondary/Hospital nurse 1 Nurse specialist in stomatology 1 Expert nurse in digestive 1 Nutritionist 1 Nursing assistant

### Data collection

The relevant data were collected via in-depth interviews held between January and June, 2014. Once the participants were chosen, a member of the research team contacted them via telephone. During the call, the characteristics of the study would be explained and the candidate would be invited to participate. Appointments were given to those agreeing to participate. The interviews were held in the healthcare centres of the participants’ choice, where an appropriate level of privacy and confidentiality could be achieved. Furthermore, the time of the appointment was adjusted to the participants’ preferences. The interviews were approximately 50 minutes in duration.

### Data analysis

The principal investigator (SGS) guaranteed the coherence and precision of the data with regard to performing interviews. The interviews were recorded with a digital recording device, transcribed textually and subsequently presented to the participants in order to substantiate the accuracy of the transcription. The data was processed via a qualitative content analysis approach [20]. The interviews were analysed by the authors via the triangulation of data, applying an open and inductive codification system which involved assigning emergent codes to each paragraph or sentence, which summarised their meaning. These codes were classified into groups according to similarity. Subsequent to identifying patterns in the interview transcripts, classifications were divided into topics and subtopics. Once having identified, analysed and contrasted the possible differences with regards to the available bibliography and/or conceptual frameworks, the content was then further screened and consensus reached concerning the most relevant data making up each topic and subtopic. Trustworthiness of qualitative data was achieved via the systematic process of both data collection and analysis [21].

## Results

Three (3) topics and seven (7) subtopics were generated. The topics and their constituent components or subtopics are displayed in Table 2.

**Table 2:** The topics and their constituent components or subtopics.

**Topic 1: The healthcare system as a hindrance for ongoing treatment of CD**	Subtopic 1.1: Lack of connection between different levels of the healthcare system (i.e. primary/community and secondary/hospital levels). Subtopic 1.2: Dehumanization of professional healthcare assistance.
**Topic 2: The impact of the disease**.	Subtopic 2.1: The moment of diagnosis and the information provided. Subtopic 2.2: Private and public life with CD
**3. Topic 3: Support networks**.	Subtopic 3.1: Family support as a fundamental cornerstone. Subtopic 3.2: Lack of professional support from a multidisciplinary team. Subtopic 3.3: The search for mutual support.

The topics found were as follows:

### Topic 1: The healthcare system as a hindrance for ongoing treatment of CD

#### Subtopic 1.1: Lack of connection between different levels of the healthcare system (i.e. primary/community and secondary/hospital levels)

The study participants reported breaches in the continuity of care, above all between primary and secondary healthcare professionals: Interview B in S1 File. “*the patients are closely managed from the digestive specialist unit* (secondary level)… *they don´t trust us at the primary healthcare centre”; Interview E in* S1 File. “*There are times when the General Practitioner (GP) does not wish to follow patients up and they are constantly referring them to the hospital”; Interview I in* S1 File. “*that´s the problem*, *they don´t wish to go to any other doctor except for the one who diagnosed their illness*”.

Furthermore, it was expressed that there is no consensus regarding the continuity of care and/or the monitoring of CD. In fact, the information provided by the healthcare professionals was on occasion contradictory, which is disconcerting for patients: *Interview E in* S1 File. “*it´s as though colleagues at the hospital do not delegate enough for patients to gain confidence in the professionals at primary healthcare centres”; Interview A in* S1 File. “*they are very demanding people and they come to the hospital for any reason*, *even issues that could be resolved with their GP*”.

#### Subtopic 1.2: Dehumanization of professional healthcare assistance

In the words of the professionals themselves, a dehumanization of healthcare assistance can be foreseen, since they are aware that Crohn´s Disease is a very complex illness which should be treated by a coordinated, multidisciplinary team and not by isolated individual professionals who treat a particular aspect and lose sight of the holistic perspective: *Interview C in* S1 File. “*You go to see the digestive specialist at the hospital and perhaps last time you were seen by a different one and they review your treatment and that´s all*, *having a reference point is extremely important”; Interview F in* S1 File. “*There is no time for more than treating the current bout … perhaps if they are depressed*, *you prescribe a sedative … but not normally*”.

Further to the aforementioned, the healthcare professionals defined people with CD as very demanding patients who require much attention. This leads to healthcare professionals erecting barriers to people affected by CD, due to their lack of personal experience: Interview A in S1 File. “*there are people who have followed me from Madrid … these are patients who pursue you over time*, *they call you by telephone*, *they even want to invite you to their table*”; Interview D in S1 File. “*they also expect you to be further involved*, *due to the nature of their illness and their interest in controlling it*, *they tell you what you can and cannot do*, *casting doubt on your professionalism*”.

### Topic 2: The impact of the disease

#### Subtopic 2.1: The moment of diagnosis and the information provided

The healthcare professionals highlighted the fact that patient’s do not adequately assimilate the information provided at the moment the diagnosis is communicated to them. Thus the decision is made not to provide the information in its entirety in the first instance, whereby a protectionist attitude is adopted. Simultaneously, the patient senses the severity of the situation and experiences a lack of control: Interview H in S1 File. “*Well*, *in the first instance you give them the information but they don’t assimilate anything*, *it’s enough having to comprehend they are no longer healthy and will have this illness the rest of their lives*”; Interview G in S1 File. “*They slowly take the serious nature of the illness on board*, *it´s part of the process*, *but in most cases they believe they are going to die and until they realize it can be controlled with medication*, *they are very scared*.**” It should be noted that the autoimmune component of this illness finds patients feeling they are initially lacking in information and control over the disease and as a result start constantly searching for sources of information: Interview B in S1 File. “*they arrive at the healthcare centre very well informed*, *either by their doctor or via the internet*, *in fact sometimes they ask me about things I am unaware of*”; Interview I in S1 File. “*by the time they get to me*, *they are well informed*, *these days you surf the internet and you find out about everything*, *yet they are not always gathering the correct information*”. Moreover, reference was also made to how people with CD, on occasion, acquire an excess of poor quality information which does not always comply with reality and becomes confusing: Interview J in S1 File. “*they are always looking for information*, *on the internet*, *by talking with other healthcare professionals*, *with other patients … and sometimes it´s worse*, *because this illness is not the same for all patients*”

#### Subtopic 2.2: Private and public life with CD

The health professionals were conscious of the obstacles having CD involves on a personal level: Interview J in S1 File. “*if you are having a serious bout*, *you end up in hospital and of course this interrupts your life completely*”, on a professional level: Interview F in S1 File. “*this condition makes you dependent on always having a bathroom close by*, *in fact*, *I always recommend my younger patients study a degree in order to work in an office and have a bathroom nearby …*”, as well as on a social level: Interview H in S1 File. “*sometimes they explain they can´t go out*, *that they feel unwell … and at that moment you consider referring them to a psychologist*” or even on a maternal or reproductive level: Interview A in S1 File. “*currently people are delaying the age at which they marry and have children since they find themselves with this illness and say to themselves “wait and don´t fall pregnant …” and then they say “gosh*, *I´m 32*, *my biological clock is ticking … wait for now*, *there´s no way” and then there are cases where the pregnancy must be interrupted because the illness has taken a turn*”.

### 3. Topic 3: Support networks

#### Subtopic 3.1: Family support as a fundamental cornerstone

According to the healthcare professionals, the greatest support people with CD have comes from the informal realm, precisely that received from their family and surroundings: Interview C in S1 File. “*support basically comes from the family and as they are young patients*, *from close friends*, *in which they can … We are talking about very young patients who usually arrive accompanied (by someone)*”, Interview K in S1 File. “*they always come accompanied (by someone)*, *at the beginning and throughout*, *in fact the family even ends up knowing more than they do*, *they become very dependent patients*”.

#### Subtopic 3.2: Lack of professional support from a multidisciplinary team

The majority of the professionals interviewed coincided in the idea that the best manner to attend to patients with CD would be via a multidisciplinary team: Interview I in S1 File. “*a team would be ideal*, *but we are talking about very extensive services with stomatologists*, *gastroenterologists*, *specialised radiologists and surgeons*, *nurses completely dedicated to the issue and also*, *of course*, *a psychologist who provides support in moments of emotional downturn*” Interview J in S1 File. “*it would have to be a multidisciplinary team with the primary healthcare centre and the hospital interrelated … it should be a multidisciplinary team and*, *of course*, *involve the family*”, albeit they recognize this is very difficult given the current state of public health in Spain. Several of those interviewed remarked there were isolated efforts to make such teams a reality: Interview H in S1 File. “*in such a way that there is a person connecting the team at the day hospitals*, *they are a fundamental figure since there is always a telephone (number) to call*, *always a person such as those at the day hospital who filters the calls and is there to provide support and make visits amenable*.**”

#### Subtopic 3.3: The search for mutual support

The healthcare professionals interviewed perceived the existence of an important support system on an informal level among those who find themselves in the same situation: Interview K in S1 File. “*while they are receiving treatment at the day hospital*, *they are always commenting on their experiences among themselves and they give each other advice”; P09 “they speak to other patients about different treatments and options*”, as well as on a formal level, via associations and support networks: *Interview F in* S1 File. “*once the diagnosis is established*, *I recommend ACCU to them … although most of them do not become active (members)*, *they do like to be informed*”; Interview K in S1 File. “*I founded a group on Facebook and they talk about what´s happening among themselves and they feel supported*.**”

## Discussion

Qualitative evidence shows that during the healthcare professionals’ academic training, knowledge of CD in terms of the real-life experiences and psychosocial aspects affecting patients and their immediate circles, is mainly gained experientially. This evidence has been confirmed after reviewing the health professions’ study programs in Spain in which few hours are dedicated to CD though only from a clinical perspective. Moreover, there is a lack of specific contents regarding psychological and social aspects of the lived experienced of those affected. This leads to the suggestion that it is a topic barely touched on in the academic syllabus and depending on the healthcare professionals’ experience in the workplace, a greater or lesser amount of information will be available. As a consequence, it is difficult for healthcare professionals to know what life is like for people with CD and as such, to quantify its impact on their lives.

A number of barriers or limitations have been expressed by healthcare professionals with regard to understanding the experience of those affected by CD, such as matters of the coordination between primary and secondary healthcare and a certain dehumanization of healthcare services. Coinciding with other studies [22–24], an effective system of interdisciplinary collaboration between primary healthcare and secondary or specialised healthcare professionals should exist for a better coordinated service for such patients, with well-defined clinical practice guidelines (CPG) and clinical pathways, which might allow for an integrated healthcare system. The national healthcare strategy for chronic diseases [25], calls for the creation of CPG in order to guarantee effective and safe healthcare intervention, as well as tools for facilitating decision making processes, for both healthcare professionals and patients affected by chronic conditions [26], yet there are no specific references to CD, as opposed to other nations in which ongoing improvements to the CD patient pathway are promoted based on consensual advice, as is the case of the National Institute of Care Excellence (NICE) [16] from the United Kingdom. The implementation of CPG or clinical pathways [16,27], facilitates the continuity of care and achieves appropriate follow up on patients. At the same time, an increase in their quality of life is attained [28] and in the case of clinical pathways economic value and efficiency in the use of resources would be achieved at all points of the CD patient pathway from the moment they are diagnosed.

Furthermore, the aforementioned dehumanization in healthcare, as implied in the results presented here within, would need to be evaluated, whereby dehumanization is understood to be the loss of those qualities which distinguish us as human beings while ignoring individual characteristics, sentiments and values [29]. Whether this is due to the current emphasis on prioritizing objective scientific and technical knowledge of the disease, or a lack of time and/or poor organizational management, or as a coping method in order to avoid becoming too personally involved in each case, healthcare professionals occasionally lose focus of CD patients as the bio-psycho-social beings that they are [30], or due to the pathologic focus of the Spanish Healthcare System, a philosophy which centres on the clinical profile to the detriment of holistic principles which centre on the person as such [31], as pointed out by WHO [32].

Regarding the information provided to patients, healthcare professionals are aware of the importance of choosing the moment and keeping in mind how the information is communicated. There are similar studies previously published [9,33,34] that considered this moment as a fundamental part of the assimilation of information regarding CD. Others take into account the gaps in information potentially created by using overly specific language [22,35], as well as the differences in expectations between professionals and patients [11,33].

The results here presented show that people affected by CD manifest a strong concern for being well informed. Perhaps the information offered verbally could be accompanied by health education and the promotion of well being and not as an isolated issue [36]. The results of this study uncover a communication based on a mere exchange of clinical information [31], far removed from the concept of health education and therapeutic advice exploring aspects such as lifestyle and habits which would require further specialization on behalf of healthcare professionals.

It is evident that healthcare professionals have a limited understanding of how CD impacts on the lives of their patients [22] and this can act as a hindrance to the integrated ongoing care of CD patients, despite knowing the limitations this illness can impose on many levels such as family (having children), work (obtaining/holding down a stable job) or social aspects (limits to leisure pursuits) [37]. Occasionally, healthcare professionals are capable of detecting psychological problems, yet are limited in their capacity to treat them since they must be referred to other professionals who are external to their division [12].

These results coincide with those of other studies [38,39] in that healthcare professionals consider informal networks of care, the CD patients’ immediate circles, are an extremely important resource through which the latter feel supported. They represent a further channel of communication with patients since they are constantly in touch with healthcare professionals and eventually come to understand the disease to an equal, if not greater extent as the patients themselves [40,41]. They also realize the importance of associations of patients with CD and self-help groups belonging to social networks. As shown by a number of studies, it is comforting to be able to share one´s experience of the illness with others in equal or similar circumstances [42–44]. Again, the learning derived from the patient’s real-life experience of the disease is translated into the gap that separates them from the healthcare professionals. Consequently, it is the patient who eventually takes on the role of an expert, via the empowerment acquired from the experience and information received from other patients. This role as an expert is naturally conceded to them by the very professionals themselves due to the aforementioned gap in real knowledge they possess [9,45].

As described in the available literature [13,46], multidisciplinary teams would constitute the most appropriate means of attending to people with CD. The results of the present study show the healthcare professionals involved are concerned by the absence of such teams. Were such teams to exist, the quality of life of patients with CD would increase, obstacles between those affected by CD and healthcare personnel would be eliminated and the integrated care of CD patients would be facilitated [47,48]. Nonetheless, there are some Spanish hospitals [49–51] where efforts are being made for such units to become a global reality.

## Conclusions

The knowledge of CD gained by healthcare professionals, in the contexts studied here within, with regards to the psychosocial aspects and the experience of those living with the disease and their immediate circles, is poor, if not null on an academic level, becoming experiential on their incorporation into the professional field. Additionally, a priori, they lack the tools to address the doubts and concerns of patients from the moment of diagnosis through the ongoing care of the patient. Organizational hindrances, such as the lack of time and consensual guidelines for adequately monitoring CD patients in Spain, further restrict the patient-professional relationship. Therefore, it can be said that healthcare professionals have a limited understanding of the impact of CD on the day-to-day life of those affected, not being considered a part of the CD patients’ formal support network. Nonetheless, they are conscious of this limitation and advocate for multidisciplinary teams as the best means of attending to people living with CD. Our study outcomes may represent the first step onto identifying strategies and best practices for establishing an effective therapeutic relationship, as well as any hindering factors.

## Research limitations

The data gathered reflect the experience of healthcare professionals from the province of Alicante, Spain, as well as particular healthcare institutions, thus they cannot be extrapolated to other populations that do not possess similar characteristics. However, the type of participant and context selected in this study can be considered as highly representative of the experience lived by CD people and professionals in Spain. Even so, the geographical limitation should be addressed in future research with similar approaches in other provinces of Spain, particularly so given the decentralized nature of the Spanish public healthcare system and differences to the private system. Although information saturation was reached through the interviews and despite statistical sampling not being a requisite for qualitative methods, further research into the viewpoint of other professionals in a range of contexts is essential in order to shed light on the status quo in Spain and to assess the approaches being applied by the healthcare system.

## Supporting information

S1 File Interviews A-K. (DOCX)
