# Supplementary material for: Healthcare professionals’ views of the experiences of individuals living with Crohn’s Disease in Spain. A qualitative study
Source: PLoS One. 2018 Jan 23;13(1):e0190980. doi: 10.1371/journal.pone.0190980 (PMC5779654; doi:10.1371/journal.pone.0190980)
Supplement: S1 File — (DOCX) [file pone.0190980.s001.docx]

S1 File

This file does not contain personal or patient information and ensure that the data shared are in accordance with participant consent.

This study was approved by the Ethics Committee of the University of Alicante (Reference number: UA-2016-06-20). All participants were informed by the researcher of the aim of the study, the methods used and how they would participate. Prior to being interviewed, informed consent was obtained in writing from those participating, as well as the acceptance from the institutions to which the personnel interviewed belonged to.

The support information shown below corresponds to the textual accounts that are shown in the results of our studies, each interview is named with letters and the corresponding number according to its order of appearance.

**Interview A**

**Specialist doctor in digestive system 1**

**Hello, the reason for contacting you was because a nurse who worked with you in the day hospital, attending to people affected by the EC, told me that you had mounted a very special unit to treat these patients ...**

Yeah , well, there have been many that have been made in the context of day hospital, there was a study, but hey this was a nationwide study was the study CACHE[http://geteccu.org/investigacion/proyectos-finalizados/ study-cache](https://translate.google.com/translate?hl=es&prev=_t&sl=es&tl=en&u=http://geteccu.org/investigacion/proyectos-finalizados/estudio-cache) , which had to do with how considered patients was the attention that had supplied them and now we have a project that is granted ascholarship to a psychologist, you are measuring the degree of stress that patients have and then goes do ... that 's going to be your thesis and then she then will make an intervention group because it hasworked well in group therapy in patients with morbid obesity, which is involved and oncology patients, and had interest in creating the group, but there was no money in the hospital or was hired by the hospital, then we got an industry grant, because we had no other way to do it and in principle now it is in an initial phase of a measurement e stress level of patients, passing stress scales. And then what he's going to do are two groups, one with intervention and the other without intervention to see how they evolve, to see how things change. This is the project that we have a little more in this sense, the psychological aspects and the quality of life.

**What do you think is** **more** **q** **ue a person hits you** **when** you **receive a diagnosis of Crohn's disease?**

The impact is that of any chronic disease, let's know that you have a disease that is forever and that is not going to be cured. In principle you have to accept that you are sick, when you go from a healthy to a sick situation you have to assimilate it and also that it is a chronic disease and also that you have to transmit the truth and that it is a disease that as its specific cause, although it knows a lot about the pathogenesis, there is no single specific cause, it is unknown, as it does not happen with the infectious diseases that are caused by a germ and when it is treated, with an antibiotic or antiviral treatment they are cured, this is another totally different concept. This is the "I have a disease that has not been discovered so far exactly what produces it and I'm not going to cure" but then comes the positive part is that yes there are treatments and in the near future many more, they get , to keep the symptoms controlled and for the patient to lead a practically normal life. What happens is a disease and this is told from the beginning, which is a very heterogeneous disease, this is another thing that defines severity in a patient Crohn's variabilísima, there are patients who do not learn that It has it and there are others who come to have a risk for their lives and come to have a total colectomy and live with a stoma forever or with a reservoir via anal, which also has its consequences, that is, in the case of the EC there is a small percentage of very mild forms, but it is there of people who can be maintained with salicylates even people who need biological treatments with potentially very serious effects, although many of them are well maintained and other patients who will require interventions and not only one but more than one throughout his life. So the spectrum is very variable, this also has to be transmitted and then a little because that, which has advanced a lot in terms of surgical techniques, also in the treatments and therefore the prognosis of the patients is very different, since many of them have a totally normal life

**Normal?** **Once the impact of the diagnosis has been overcome, what strategies do you use to reach that normality that you mention?**

Well in the case of young patients, adherence to treatment, because having, you are young, you have a very concrete life, with many activities and others, having to remember to take the pills or depend on the hospital to come and put on a treatment or if you are at home having to put it on, because we are talking about injectables too, all these things, because they ... are difficult for patients. This is what changes them a bit with respect to normality. And let's not say when the course of the disease is more torpid and forces them to go to the hospital on many occasions, because things are not going well, or hospital admissions, and so on. After the initial impact, as it will vary greatly depending on the course you have, that is, depending on the severity of the patient are talking, which ... is upside down, the patient then follows a mild course says *"uy because with what I worried at first, it turns* out *that I* live *a normal life, I take my drugs* , *"as* we say often is as hypertensive, diabetic, with the difference that this is a disease of young patients and sometimes take Engente young medication because, this has to be convinced. And then there is the poor guy who has bad luck and he has the most torrid course, because he has to get used to it. Then they have a lot ... it is a disease that affects the intestinal habit and this many connotations for patients who are school age, because having to leave class, to use the bathroom, and not to mention if the bathrooms are nearby and think that there may be escapes, smells, etc., because all these things affect them psychologically. And then the abdominal pain, because it makes them feel uncomfortable or feel ... this is what the patients in the surveys say that have been done in the patient groups, in European surveys, because the patients tell it very well, they feel that it affects his academic performance, his work performance, his interpersonal relationships, that have left relationships, with divorces, with ruptures ... The patient who has many difficulties, because there are many people who are not able to support and follow the path and look for the easier ways, I refer to couples and this is true that there is a very high percentage of patients who refer all these difficulties that already affect the emotional sphere, but are totally linked with the symptoms of the disease.

**Who are they seeking support?**

Basically in the family, and also as they are young patients in close friends, in which they can ... We talk about very young patients who always tend to be accompanied. And then with the day hospitals are a fundamental piece because there is always a phone to communicate with, there is always a person like the day hospital people who filter all the calls and who is there to give support and who allows that , that visits are always close, we filter everything so that it does not delay, because this works like that and of course at the end of the chain, we doctors.

**Who do you think should be the right professional to follow up?**

The teams, when trying to improve patient care that there are psychology units that are, but implanted within the team that would be ideal, but we are talking about very large services that have stomatherapist, radiologist and specialized surgeon, nurses totally dedicated to the subject and also, of course, a psychologist, who will support them at the time of that downturn. Here, a psychologist who got a private scholarship and is conducting a pilot of an experience, but not part of the unit itself, we do not have an assigned psychologist, have a psychiatrist who is part of the whole hospital and that he you caninterconsultar. Here there are no beds of psychiatry, then everything works with interconsultations, but really the sick then, you also have to recommend that they ask for an appointment in the area mental health units, but it is not the same relationship you can have with someone of this unity. And of course, sometimes if that antidepressant treatment is necessary, but sometimes not, with psychological support it is sufficient.

**Interview B**

**Specialist doctor in digestive system 2**

What do you think about the impact on a person of receiving a diagnosis of CD?

Fear, why fear? Because it is that fear of the unknown, that is, when you tell a patient in a certain way that ... first when you ask for the diagnostic test why do you ask for it, when you have the diagnosis and you explain it, fear, because he does not know what it is. Even if you explain again that it is an autoimmune disease, that it can evolve into outbreaks, that it is a disease for a lifetime ... it is fear. Maybe not the disease itself, but the unknown, because it does not know what it is. A gastric ulcer knows what it is, a peptic ulcer also because it has heard it in a certain way, but inflammatory bowel disease ... it sounds more like ulcerative colitis than not EC. Because the neighbor has it , because Perico of strokes has been hmm ... but the EC does not quite have it clear.

In which persons or group of people does the affected seek support?

In the family. It is the family because they do not come alone from the first moment and when you realize they start or that some of the members are in the sanitary part mmm, they begin to open their eyes, but always the part of opening the little eyes is not so marked, because they also do not know what the disease consists of. But the main support is family, at least from my point of view.

What are the main difficulties, which in your opinion, face those affected by this pathological process in their daily lives?

Chronicity and recurrences. When you tell a person that it is a chronic disease, that he will have to take medication all his life, that he evolves into outbreaks, that the nervous tension factor is very close to the disease, that it is an autoimmune disease, Immunity, on the part of one, they did not see it ... and fundamentally, it is chronic and the recurrences that it may have. That it can be located in a place or it can be extended. Going bad you can extend a lot, with fistulas, various locations, which can appear from the mouth to the ass, and fistulas. That's where the patient really faces the pathological process.Therefore it is chronicity and relapses.

It considers that those affected have adequate information regarding the symptoms, treatments, diagnostic tests, restriction of activities ...

I have to ask you before or after?

**After diagnosis**

After diagnosis, it depends on the doctor

**And in your case?**

I explain them from "a" to "z", when we talk about everything, I tell them this is what we are going to do, this is what can happen to you ...

**And** **do** **you think they understand?**

Well, they ask me a lot in the consultations, but I give it all in writing, that is, when I have a patient like this, I have everything ready, what the disease consists of and how it is going to be able to have it.But more than anything you ask me what is the periodicity of diagnostic tests, ie, the questions are based on the information I give them. But the total information, only 10% of patients ask me insistently throughout the consultations. And when more of age is, more still ...

What have been the main coping strategies that have been detected in those affected by CD?

What does "coping strategies" mean to you?

**Well once you know what happens to them, what strategies do they use to be able to adapt to the disease or cope with it ...**

Is it that I always remain in chronicity and taking medication, why? Because when you tell them you have to take medication for life, you first have to chutarse corticioes has to chutarse mesalazine andClaversal and depending on how you have medication gastric protection, maybe also enemas ashhhh! It is a world okay?. Now, what is it that worries the patient in a certain way? Chronicity is already clear, the mediation when, how and why. And there you have to be reiterative every time they come you have to reinforce the medication they have to take

**And** **you are able to perceive from your office chair, how** to **cope disease in your daily life?**

Mmmm no, this will not get to perceive ...

**Throughout the evolution of pathology, at what times do you need more support?**

In the outbreaks, I see them here. I see them in plant if you are my patient. Because the problem in this hospital is that the outbreaks when they arrive, if they are not yours, go to internal medicine, and who takes those patients are them. And if internal medicine can not call you. My own patients if I have, when I managed the income, but if the person comes from emergencies, will internal medicine ... well sometimes call me, although I've enterao after one of my patients I was ingresao here. The patient comes with such fright or so much fear that he does not ask for me. But all this goes in function of internal medicine, if they look for you, there are even times that if a doctor takes you from outside the clinic they let you know, but ...

Do you think that the needs of those affected by CD are fully covered, in an integral way?

It is the lack of information about the disease, that is, it will be covered, to the extent that the information needs are covered. If there is a knowledge on the part of the patient. For example, when you are admitted, you have all the needs covered, but in the day to day and in the street there is no need.

I give information, but when the patient enters he is totally covered and it is the medical treatment, the dietetic treatment, the nutritional treatment if it is necessary, of course ... but beyond it he does not leave.

What do you think could be done to receive comprehensive care?

More information, we always return to the same, the information campaigns of the disease ...

**What do you mean when you talk about information, treatment, risk factors, diet ...?**

But to be consulted ... Just as there is a group for coeliacs ... there is also Crohn's, but is that these people do not know why? As we are crushing and you will see that on my Facebook there are different things, but fundamentally obesity, obesity, obesity, that is to say they are colon cancer campaigns so that people become aware, they are stimulated, talks are made, workshops are created ... what, about a disease with a big impact like colon cancer. But of this, how many workshops have been done? Any. Now we go to the irritable colon ...

**Well at the level of this Community, because as you well know in other Spanish Communities if this campaign is made that you comment ...**

Sure, that's why I tell you, these people do not ... you have to stimulate them a lot. I always give them internet pages to consult things ... I can not give them everything in writing, it's not something that involves me so much, because I do not only take patients of this type ... I can not get involved in just one. Unless I am a patient in whom, when I have Crohn's or colitis, I go for it until the end, yes and my particular mobile phone, I can assure you

**Then I understand that the information for you would be in terms of treatments, tests, risk factors ...**

Woman that is done in a chat but I can not be stressing things, that is ...

**Not if it's not a matter of emphasizing, I understand what you say, but where are the emotions?** **Just** **information** on **a physical level?**

Yes because that terrain is already at the level of psychology, there is already a psychological profile, I can not get there ... even many times they receive a triple treatment, doctor, nutritionist and psychologist ... depending on what is coming up. This would be the integral treatment of the disease and the younger it is the more it needs, because it is more limited ...

**What do you mean, you're limited?**

Of course limits, when you have the cagarritas, you're leaving for vareta, as I say, with eight or nine daily bloody stools, to see who is the handsome tells a boy of 20 years a cubata not take ¿ eh ?, Of course it limits life in many ways ... at least from my point of view. Even now I have one of the girls that apart from Crohn had the misfortune of having anorexia nervosa, which have joined the hunger with the desire to eat, and now I'm worried that takes a few days senagrando and already have chutao everything you imagine and more, I've ingresao, you've done in the day hospital with Remicade, have made him steroids, we have made ... I do not know what more to give and Pup, again has diarrhea again … you understand me? There are very complicated cases ...

**Do you have a more personal relationship with her?**

No, because she is medicated by a psychiatrist

**That is to say, that in general your relationship with patients does not overcome the barriers of your function as a digestive**

Yes, somehow, but it's because you do not have time to do it. Look at the list of patients I have for today, as you will understand I do not have time for it, I will try to detect how it is and depending on that I will refer it to a psychologist or a psychiatrist ...

**Well, at least you detect it and you derive it ...**

Of course, detect and derive.

Who, they believe they should be professionals who would be responsible for covering possible support needs in affected?

The doctor, the therapists ...

**By making derivations?**

The therapists can not do anything, the nurses do not have access, psychologists do not have access, dietitians do not have access, if they do not come from someone. And within the doctors ...

**What is your involvement with the affected throughout the process?** **Do** **you** **consider part of your support network?**

Of course I consider myself part of their support network, especially at the beginning, because later they get used to it over time. But the doctor, but the doctor, because often the doctor when there is a good feeling, depending on which doctor makes a report, calls me and sends it ... and then we put the whole process in motion. The doctor is the fundamental part of the whole process because it is the one that is most involved in the follow-up, it is who prescribes the medication, who is constantly seeing it, but that in a joint way of working with the digestive doctor, but as long as the doctor of bedside wants to have relation with the private health, because many times we have found that a patient has arrived at his doctor with the report of here, the colonoscopia and the prescription and they have not wanted to make the prescription

**Interview C**

**Specialist doctor in digetive system 3**

**Where do you see your patients here or in the hospital?**

For man, the patient has different degrees of involvement, there are acute forms and then after a trace by ... because it is a chronic disease, then sometimes the first tacada is here (private clinic) and other times because he has attacked more severity, it is treated in the hospital, it depends on the diagnosis ...

**And at the time of diagnosis, what do you think is what impacts them the most?**

I think they do not have any impact, because they still do not know anything. Therefore in diagnosis they are absolutely ... must look at the problem as a chronic, relapsing disease, but has not yet beenenterao! So they still do not know the severity of the disease and the conditions they may have. Then , when they are having they are realizing the limitations that may have ... but input, say good "thathappens to others but I do not go" ie get the disease a little like "let that job , I have a chronic illness "but they do not give greater importance to the issue, there you do not hit the ax, do not pose any difficult situation or anything. It's when you start telling them about the treatment, the side effects of the treatment and when the disease comes back. Then it is when they have really aware of what they have Contao,who have a chronic disease, and say "Leches! That the disease is here, and the disease returns "eye that they have given me a medication, that is making me grow hair, that is making me have a high tension, that is making me eat more, that is making me very nervous , in short, a medication that is producing a series of things ... and that is when they begin to realize what it is that you are handling, the problem with them

**Do you perceive limitations in their lives?**

It is the outbreak of the disease, the outbreak and the severity of the outbreak, that is, the outbreaks can be mild, moderate, severe and clear, if you have a severe outbreak that takes you to the hospital, they are supposed to take you away from you. normal work activity, your normal life, then when you have the outbreak what is going to make you dependent is to have a toilet next to you because you have diarrhea or because you feel bad and good the gut does not respond as it should ...

**How do you continue the evolution?**

Well, in principle, they do not quite believe the problem and then when they see, that what you have told them is what comes to them, because they begin to assume what the disease is and what is the role they have to do. Be strict with the treatment and follow pal

**Do you think it's hard for them to adapt?**

They are difficult to adapt because it means ... although the medication is established as more comfortable and easier to use, it means nothing more or less that you have to take some pills that you did not have to take before and the fact of having to take some pills that you can have There, then, you can take them one day and the other too, but to which you neglect, as nothing happens because you do not take them for three days ...

**Do they come to tell you about those changes they make in their daily life or is it more a strictly professional relationship?**

They pose them, they pose them ... you have raised them before anything else "you have a chronic disease" and when you have a chronic disease there you have different ages in which you move, then you have a young people and then you tell them "Hey kid, listen girl, study because working on a scaffold is going to be very difficult to find a bathroom, hey girl studies, because working on a physical activity is going to be very difficult to find the toilet, hey girl studies that those who have studies have offices and have toilets next "then try, when they have the disease and are young, you try to clarify that the disease can condition your life, and that your life is badly conditioned working in a physical activity, however in an intellectual activity probably have more options to be able to solve the problem and the indirect conflict of diarrhea

**For you, the biggest problem of adaptation is diarrhea ...**

Yes of course, the gut pains are coming ... it goes in connection with the disease, the symptoms of the disease are abdominal pain, fever and diarrhea and then it has many more extra-digestive but those that are really sending, and those that give you they demand in the assistance, usually that is the triad of symptoms, abdominal pain, fever and diarrhea and if there is fever and there is diarrhea there is an outbreak, and if the disease is inactive because there is probably no outbreak or there may be less silent manifestations of those who have the disease. And then there are the extra-digestive manifestations that fortunately, contrary to other Saxon countries we have less extra-digestive manifestations, fortunately we have few, we have dermatological manifestations few joint manifestations and very few mucosal affectations, we have few affectations. Then there are other manifestations that are very uncomfortable such as perianal disease, this conditions a lot because they throw pus continuously, the problem of incontinence and sometimes the problem that you have pain permanently and this conditions them more

**These conditions are perceived by you, it is not because they tell you their limitations ...**

They do it, in fact they do it all, they are clear that the most important thing is to stay active, so they fight to overcome the disease and to have activity. They are very compliant people, they are very strict people in their work and they are people who want to live a normal life, because not living a normal life means that the disease is winning the game. So, in spite of having perianal disease or having such, they try as soon as possible to do their activity, because it is the expression that the disease is controlled ...

**So, for you, they are people who respond to the same pattern ...**

Not only that, but they have peculiar character and personality traits all of them, they are very very meticulous people, they are very picky people, very looking inside, they have very clear personality traits. What happens that it becomes difficult to know if these traits are prior to the disease or are later, because of course you do not know if the disease ... Man you notice changes, because when the disease punishes you, is behaving badly with you, you end up pissed off against him world and against all, to see why you have the disease and not your neighbor has clear, I want to tell you, that my neighbor has it that I do not like ... then they end up getting angry against the world and against everything ...

**You have told me about the side effects of the medication, does this also limit them, do they also worry?**

They do not worry, only when they have them, that is, there is not a level in this country to anticipate or anticipate what is going to come with the disease. This in this country does not exist, people do not think about it, they think about it when they have it over ...

**Do not you think they think about the future?**

No, when the medication is needed, they use it and when medication is not necessary because they frequently quit it, then it is easy, easy, easy for 30% to have an erroneous treatment compliance. But of course we talk about chronic problems and taking a medication constantly and continuously, which has to tire us a lot, because those who do not use it or we do not use it seems to us to be very easy, but to set a pattern of Take some pills every day "Milks!" ...

**So do not think you're worried about coping with the side effects ...**

When they are produced, that is, I take this pill and the sun crushes me for having hypersensitivity ...

**Do you inform them of all the side effects?**

No, not in addition to the group of drugs that we use, it has a list of such important side effects ...

**Neither of the corticoids?**

What happens, have a reading that are side effects that are very quiet, very silent, only when you see the side effects, it is because they are already, you have broken your bones by corticosteroids, because you say "what a job" no? , the corticosteroids, who was going to tell me that what I was taking was going to produce osteoporosis and now I have a fracture of the femur neck, and you do not think about it. "My face is getting fat", yes, but I have the disease controlled because I have no symptoms and the face goes to a secondary level. "Hey you're going my hair" and pisses me leave me much hair steroids, then it is clear, it pisses me off a lot. But it's what we have, then you get to the next step, the next echelon of medication ... "but that azathioprine gives me pancreatitis" and yes you say "Fuck the milk, as this really is a big problem" but Of course, we can not continue there, but the side effects of the drugs are not questioned from the beginning, but when they are there, when they are over them

**Who do they support?**

In the family, the family, the fundamental and logical support is the family. And then they have a situation that is very rare, they are very rare people, they trust who they trust ... and normally the one who has first established the diagnosis, they trust in that for life. A link is created "He knows what he had and this goes a long time that they did not know what he had and he knows what I have" and then they create a very close link with the doctor, but with the one who establishes the diagnosis ...

**Yes, because they are also late diagnoses ...**

No, it is variable because the disease has two behaviors, in a very silent way and then it depends on the countries the average time that is established between the first symptoms and the diagnosis is very variable, as there are countries that establish a time of 10 years on average. There are other times that the disease is diagnosed in the first outbreak, because it has a lot of sharpness, because it is very cruel and because it is very active, if the outbreak is very strong and has very bad milk because then you diagnose it by throwing mistos, there is no It poses no problem, because the diagnosis you establish at the beginning, but it is variable. Then there is also a great overlap between the symptoms of the disease and functional disorders, you may even be looking for a Crohn and you can not establish the diagnosis because you do not have enough parameters for the diagnosis ... then you say "I suspect you have a Crohn, but I do not have enough parameters to establish it, clearly, then I better stay still and I follow you and I see the evolution "and after two, three years the manifestations of the disease begin to appear more clearly, and that's when you establish the diagnosis . But there are many who spend many years there, with the colgao diagnosis because how the spectrum of the disease is highly variable, as there are obviously some who do not establish diagnosis ...

**When you talk about the relationship with the person who diagnoses, I imagine that it is that person who then follows up, right?**

Seek them, they are seeking accommodation that has pegao the axing of the disease, it is, as you put you treat them first and they improve, from there, are dependent ...

**Do you think that your patients are dependent?**

But dependents of all, it's actually ... if you let hook a little bit you sit at the table at home, which no, no, do not allow ... "is your disease, I treat it but to me you not me lees "

**That is, they also seek support in the medical**

But the fundamental support is familiar ...

**Aha , but not only seek medical treatment for the subject and that**

No, not for the selfishness they have, you seek, you seek that they can use you at any time, by pure selfishness. Even when well, all the same, they seek you. Depend mainly on the family environment, that's clear, because it is he who endures every day, because they are people with some traits of selfishness and some peculiar personality, but rely on family and as a doctor, if allowed, the doctor enslave ...

**Then I support the family because they can not rely on the doctor ...**

What you should or can afford is that you feel at your table, missing more. But I refuse to let me sit at your table, or I have good relationship with them, I have patients many years, I've had for a long time at the end've been cutting a little links, people following me, I still still from Madrid, I appeared in Alicante in the year 84 it seems like 30 years ago and was sick in Madrid who came to see me here in Alicante

**Is this characteristic of patients with Crohn?**

I have no other patients who persecute me so long ... yes yes actually call you on the phone and make you a firm track, in fact I think I only have one, you must understand bad this did not make a label, this will I established the diagnosis, had symptoms long ago, we established the diagnosis and clear, this is a disease, not for the private sector, is a disease for public activity, because it is a disease that can be very expensive, and it is a disease chronic, and of course people feel there or not cobras you or you have liao , because of course if you have an outbreak and have to come every four or six weeks or eight weeks and each time it gets there, you put the saucepan and pum, pum pum and pum (Gesture payment on the table), is a tremendous drain, then tell them to be a disease of hospital follow-up, why, because if you need hospitalization, hospitalization is very expensive and then I only saw one that I said "everything is fine, you open the door, but I recommend you go to see your doctor to send you to your specialist and have a reference there that diagnosis. I see all the time it takes ... "He got angry and did not return, it was as if he had betrayed. I have not betrayed, I've told you to look for a hospital, if the disease lacks a hospital, and of course the disease itself lacks a hospital and of course, this interpreted it as I wanted me remove above, God forbid,I'm trying right now 200 and peak 300 Crohn's patients, in fact I have sick, I do not know why, I came once Cartagena and have a colony of Cartagena patients who come to see me, but I also have a military that is in Cadiz and from Cadiz to come see me shit you do not need you to come! There also can see you ... "I come not ..."

**Let a link is created ...**

It is the only patient I have, I have let many sick and is the only one that creates a link after you, to follow ... are these. They establish a very narrow, very dependent link; and in fact inflammatory disease units intended to make that coverage always have someone continuously where they can turn to, without having to ask what a horror quotes, by God !, just eating and picnicking with crones, and that can not being, the profession is one thing and private life is another, then I have very good friends that I relate very well with them and already know them very well and what I say is the word of God ... but I think you have to disassociate affectively professional issue ... because we them, they will seek dependency, we seek ...

**Does this also happens with the family?**

With family are worse, the family have puteada. Because it's the one ...

**Both in remission and exacerbation ...**

Anyway, they are selfish and they are suspicious, selfish, distrustful ... people with very peculiar features are rare, in fact if you investigate causes of disease always talk about personality traits ...

**Yes, it's true, we talk about psychological traits ...**

It is a disease, you probably have a mixture of autoimmune and infectious is not clarified, but there is probably his action, indeed What answers? For the corticoid and what the corticoid does it reduce immunity, if an infectious disease in the classical sense, lower immunity significaa the disease still going to explode again, it is a fertile field .. then it is closer immunity probably

**Why do you say that family is worse?**

Because they are the ones who put up all year. Is the family that comes with the disease is that it is the mother who comes with the illness of a child, it is that it is the mother who tells me the child's illness ... is living the illness of a child, husband or partner with such intensity that the disease but fully known, and know when it was taken and the pill are aware of them and they know they have an outstanding person ...

**How kind of supervisors?**

No, no, they are become enslavers of the family network, but that, exactly. Then of course, it can not be that a guy of 40 or 50 years have to bring the disease to others. In the other diseases I do not matter, or other problems and no family members take care of the disease. I see diabetic and diabetic manages himself and is a chronic disease, I see patients who have Parkinson's and they will depend on the neurologist, they will raise the dose but do not have the family member who best knows the disease he…

**Why do you think this happens?**

Because they are, because it is their way of being, it is his nature, is selfish people, it is peculiar people, it is people who have a ' tocao ' and that ' tocao ' is what makes them do unto others have them at your service. As happens with the doctor if left have sentao to your table, same, same, same, he spent with family, but you are can afford it or not, but if there are people who need to rely on him, that carry the disease

**I understand, do you think you have enough information?**

Who want to have, I at least the part that touches me, have all they want. I initially did not Loes hit all the axing of disease problems, but at this time, the people we see inflammatory disease, we know exactly what is there and we have sufficient information to inform them of what there is , then if someone does not He knows about the disease, because it will not inform, but usually the people we see Crohn, we know what we are talking about and inform them. Here everyone has their own style. What I do is that I inform parts. You can not tell a guy who comes, which is punishable liver, pancreas, heart, lung ... then get out of here escandalizao "But I have, by God, I die tomorrow," then you go for releasing a part and as you keep watching because you're telling the part of the disease. And then later, as I always refer them to the association, ACCU, refer to them and tell them "do not have to become a member or have to go ..." magazine that issue is a sensible magazine does not say barbarities, he does not tell lies and puts you directly with the problem ... but what happens, that those who are more implicaos with the disease are more implicaos with the magazine and I tell everyone, "you have your Crohn, but I have 200 or 300 crones then I have more knowledge of Crohn than you, therefore I have more knowledge than you, therefore let's make things clear, to see what we know each disease "but I command the magazine,when the diagnosis is made, I will command to ACCU ...

**What do you have when you have gone to ACCU?**

Which will ACCU two, some who convert to Catholicism and have them more faith than anyone, will be his way of being, and others who only read the magazine and others who say "UUUUUU there, there are very bad people, not I have to hang out with those, I'm not as bad as those "then there are people who dedication to the cause, for the common good of society and save the world. And then there are others who say "quiet ! I with these people I do not go, those are very Pochos and that's not my problem "Behold , the two reactions. The majority, I have been once but do not return, do not fuck with me! What is there, but in ACCU are the most committed, because they have committed and the disease is not clear that. And indeed, the whole Crohn's not invalidating, in fact Crohn's little invalidating. But of course that is invalidao , it is in the association metio but to the bars, and of course when you get out and see the calico, because people do not mess. It is not a sect but ...

**Going back to the issue of information do you perceive that they understand all the information?**

Man, this country has the level you have, let's see, the level of illiteracy we have is the level we have, there is no turning back. Illiterate operating in this country are many who cuandoyo I speak How many understand me? Well I try I understand everyone, but certainly not understand me all because between what I say and what they are blocked and they do not understand, well maybe the level of information they end up receiving, is not the I have them conveyed But I do not, this is business as usual. Not because I tell you five times what you'll learn more or better. But the issue I have it very clear, and always say "I told you twice, as I tell you four I'm going to say like the first two, so I'm not going to say more left you clear? "and give no more turning back, if I've told you twice and you have not heard, because I tell you that more times, you will find out the same thing. So, for that matter ... because otherwise there is the cultural level of this country, let you know what you say ...

**Not even young people?**

Anyway, the level of school failure in this country is tremendous and education ... if we are the penultimate country in the world in reading comprehension what are we talking about? ... if we were talking football, everyone understands, but if we talk reading comprehension, we say that there is a very high functional illiteracy

**You've told me that the units are responsible for inflammatory disease that those affected can go without any appointment. In your opinion, what would be the appropriate professionals to monitor patients?**

Ah! No, there's no infrastructure country right now, you can afford to have the situation ... I units ...

**Well, if I were in your hand ...**

Inflammatory disease units, consisting of doctors, nurses and psychological support ...

**A psychologist, do you have often susceptible to psychological treatment?**

Mmmm , it depends on the seriousness of the problem, eeeehhh If you have a rectovaginal fistula and prevents you from having sex with your husband, because you're throwing shit pussy, talking bad and soon, it is very clear that this will be a very important for you conditioning and your husband ... then you say 'my mother who is falling on me, "if you can not get pregnant because the disease puts you upside down with pregnancy ..." is that I want to stay pregnant and I thirty not know what ... "because I feel my life right now you can not, either by medication or by disease, because the disease will be reactivated if you get pregnant ... the disease is reactivated in the first and in the third quarter actually remove them from anti-inflammatory treatment, we remove them and remove them aspirins contraceptives and remove the snuff.Then of course, now people delay marriage and delay of childbearing age and is that going and you find a disease that say "be quiet and do not stay pregnant ..." and then they say "fuck, I have 32 that I rice passes "" be quiet for the moment we have no way to do it "and then of course there are some cases you have to terminate the pregnancy because the disease has upended. Then later, it is usual that pregnancy to term, but you have to be aware that the disease may not be removedI have 32 that I miss rice "" be quiet for the moment we have no way to do it "and then of course there are some cases you have to terminate the pregnancy because the disease has upended. Then later, it is usual that pregnancy to term, but you have to be aware that the disease may not be removedI have 32 that I miss rice "" be quiet for the moment we have no way to do it "and then of course there are some cases you have to terminate the pregnancy because the disease has upended. Then later, it is usual that pregnancy to term, but you have to be aware that the disease may not be removed

**How do you act when you detect these kinds of psychological problems?**

Thus giving you a pill ...

**The medical yourself, is not the drift?**

Nooo , because fools let go ... because when you fit a sandwich of pills and are like God. Anxiolytics we all, then you put an anxiolytic, as if you see it has many symptoms that do not justify the disease because you put an anxiolytic and you stay like God, but we will if you do what you ask me is what percentage of patients have with anxiolytics ?, because maybe I say that 15 or 20%, so many? Yes, many, because there is a group of them that symptoms are not justified by the disease itself, because the disease does not have active and have other abdominal symptoms and the feeling is like, well, you put medication and pulls forward

**Do you consider yourself part of your support network?**

I would not, but I have no choice ... but I do not, I do not. I want to distinguish that I am a professional working in this, but do not want to , but I have no choice, let me call me on the phone, either, in fact if you teach the phone list you'll say "Crohn, Colitis, Crohn ... "pussy ! Let but do not want, and of course did not refuse the call, of course the answer ...

**Then yes you have an implication beyond professional**

Sure, but that 's what I refuse, I refuse this to happen ... but you're going to do, you can not tell a guy who has Crohn, "I see you in three months" can not, then the guy who has diarrhea and do not you know what, you can not say "I see within not know how long" you know?

**Let you can not refer the doctor**

The problem is that they have, they do not want one, they want which diagnosed, fire is always the same, ie, they have a significant dependence who established the diagnosis and gave them the first treatment. And then as they are selfish, you know perfectly well that when you touch the key, you're gonna get treatment, you're going to change, are you going to change ... since it is already clear

**But what is surprising is that the consultation will talk about treatments, but do not have the disadvantages to live with the disease ...**

Yeah, yeah ... I'll release at least 50 times, and you have everything, you know if you have dog, if they canary ... everything, everything, engaged in studying, what sport do ... then you already you know all

**Aha , so could you tell me whether there are common strategies when coping, crushes part of the family?**

No, no, everyone is everyone, that is, there is an idiosyncrasy, there is no common strategy ...

**I ask you to wire me what you commented earlier on that share common personality patterns**

Yes, if the pattern exists ... the disease puts them in place, that is, you put bad and you take an antibiotic for seven days, but now I have problems and I can not take them, because this is the same, is the same behavior we would all, is something that comes out of my routine, and as it comes out of my routine as I slid a little away, and already, comfort or tendency to normal behavior, ie, people have pills there next to the breakfast but breakfast and take pills left, they forget, consciously or unconsciously because there are, then of course that they tell you. They ask first companion and he will die " uuuuyy has him Olvidao many times "we know them better than the patient himself, because they have made them dependent

**Interview D**

**Surgeron**

**What do you think is what most impacts a person when you are diagnosed with the EC?**

The future that awaits you

**What do you mean the future that awaits you?**

I mean, the EC is diagnosed in young people, with a disease of unknown origin, autoimmune. With an absolutely unpredictable response, because it is a disease that has no cure. It is a disease that causes outbreaks and we have medications that are improving the process, but that never quite heal, because the origin is not known. Let we can compare a little with chemotherapies that kill good cells and bad, but generally you kill them all. For the Crohn goes something like that, it's a disease by the age of presentation, young people. The possibility that it affects the entire gastrointestinal tract, from the mouth to the rectum, as if well documented patient known to have a permanent Damocles sword there for life.So ... the patient is well informed is vital, leaving the Spanish drama as we are dramatic and tragic and be aware that there is a group that goes wrong. And it goes wrong and is fatal. There are people who operas ten, fifteen and twenty times and end up dying for lack of intestine.

Then of course, what most impacts will obviously be told you have Crohn. In fact now we say "you have inflammatory bowel disease" and this is a grab-bag to see if I escape and only ulcerative colitis. To tell you today that if you have ulcerative colitis has won the lottery, because after all only affects colon and how much you planning resection with a stoma for all your life, but good heals ... but Crohn is a Crohn's disease, can affect the esophagus from the mouth, stomach ... presents with buds and you never know when it will arrive.

**I understand that you mean what most affects what are thoughts on what you can derive?**

To you they tell you "you see, has an EC" and now you can do. First to see what is my condition Crohn's sick? How I am pursuing my illness? This is Idiosyncratic, even calling themselves all, the evolution of each is individual no. That is, this is not coffee for everyone. Some evolves very isolated outbreaks and some people have outbreaks every two weeks and is the corticoideodependiente with immunosuppressants ... let that if we analyze where the treatment is focused ?. Well first shock treatment are immunosuppressants and corticosteroids, the immune response is the battle that must be waged and surgeons treat complications, which are the strictures, the abcesos or so. It is not uncommon that many start with appendicitis, or that looks like an appendicitis, then operas and it turns out is a Crohn base. It will impact you, because you say it's a thing that has no cure, you're doomed to have you shoot without you knowing when or how. That medication may not be effective, thanks to God today with what you have, they are better but time heals no ... then the uncertainty is important, because that one person will condition the rest of his life . For that you need a very important psychological support, to remind you ... and take the disease and be aware of ... the English call it "to face it "Is the face, is going to ... let's take the bull by the horns, it is that way.

**Do you get to perceive how they perform this confrontation?**

Yes, let's see ... often depends heavily on the environment, assess two things. I insist that our character, the Latin character, because we not only we Italians, I know Italy well have been in hospitals there, and are similar behavior Mediterranean, then we have a behavior trival , Ie, the disease shared by all the shares the patient, his mother, uncle, grandfather and downstairs neighbor also knows how we are ... depends heavily on the environment, ie, if the environment gives you peace of mind, that's very important. What happens is that not very typical of Spain, in general we are ... look at the history of our country are tragic, are dramatic "what a disgrace ..." Based on this, I think the environment surrounding the patient is very important . If the environment is one of tranquility, serenity, sharing things, but convey that good a thing that is treatable and such ... the patient is much quieter.

**Do you consider yourself part of that environment?**

I if man ... is always the surgeon in the environment. I mean, the surgeon are always tangential to the problem, only treat the complication, if it appears abscess perianal, the fístulita , the abscess abdominal today with interventional radiology no longer we do even that, because the interventional radiologist makes it a draw you need, and you limit yourself to operate complications. But if you're part by the type of patient, because he is young people and that makes you much more supportive.

**You collect your previous words you gave importance to information do you think you are well informed?**

No. First because it is not defined the figure of the informer, that is, we talk about information in public or private medicine. Because if we talk about public health to three minutes you are casting a because there are 33 behind you ... in my opinion I think you have to think that you have to invest time and that I can do it here in a center of private medicine, that I have my time to sit down with the patient and analyze all possibilities. Because first I give a moral reference to the patient, *"because this candidate has comforted me a little bit"*because the other site you feeling a little number, because after you come three Crohn's. Always vital, comprehensive information ...

**In your case?**

Woman, you're talking to a person who is engaged in private practice and I believe that today the great asset of private medicine, formerly the difference between private and public is that there were more media in public but today they are equal. This clinic is the same as has the hospital of Alicante, an advantage that I am my own boss and I can spend the time to me I please and I feel the time I want with my patients and my patients know who is my patient, because the other site first you have seen a small man, then another man and then has served you a very nice blonde girl. I think that in Crohn's exactly the Crohn think is important references you have. In large hospitals have a group of digestive that only cater intestinal pathology, for example you go to Cleveland and almost all patients with Crohn an area of ​​10 million people have these four professionals for them, of course they are sick who adore their doctors and not lose, do not die ... they have a specific reference. You're going to see the digestive hospital and maybe another day has seen you and you revises other treatment and it is, having a reference is important. If the question is, is it enough information? It depends on the environment depends on professional, but less comprehensive public think I ...

**Ok, but in your own experience?**

Yes and no, because we're being tangential. Let's see who tells real is the digestive, gastrointestinal specialist. Because there are specialists and experts, because it is not the same thing as you try one you like to spoil much the patient and repeat things a lot, another that although can these scientifically more prepared than the other are water and oil, so that is, by nature ... those who come to me will know everything, also today grab internet and you learn everything, and those who come to me, because now I tell you I get because they are complicated and they know that this complication there is, however have to take the complication and that depends on the environment. If the setting is peaceful, and because knowing this could happen, the options are explained and perfect ... or else is the attitude *"What Unfortunately, irreparable damage"*(laughs) Latino !

**Who do you, at the professional level, it would be most appropriate professional to support Crohn's patients?**

Undoubtedly, the digestive

**¿Only the digestive?**

Let's see, you appreciation is good because there is globalization as worldwide, what makes a Chinese, I eat your morning here, because medicine is also globalized in this regard. What would be the gold standard, it ie the gold standard. The gold standard would be that the digestive formed to general practitioners in Crohn's disease and that if in a town like Villena, for example, in Villena there are 50 doctors, because the system should allow 5 of these gentlemen were especially formed in the treatment of patients with Crohn. With this you would, because you would have within the same village "look that I have a Crohn" "Do not worry, here are 5 men who go see these days and are within their general practitioner or family, have a specialty,let specific training "for a patient with Crohn it is easier to access your doctor that digestive specialists as far you can see them once every month. So if you from the base have a person that forms properly, properly instructed by the digestive health, it is a workhorse that are in it and no way. Why the physicians are paid very little, a general practitioner who charges 1,800 euros per month and now have to formarme in this because they think *"when this patient to the emergency command turns bad"*, summarized collapse of emergency. Why what professional should be? The general practitioner with special emphasis on inflammatory disease or digestive disease and from there more or less digestive reference. Because today in Valencia this figure does not exist, Barcelona has, Vall d'Hebron has.

**Do you think the person affected p or Crohn feel well cared for ?**

Objectively if they are covered. The means that are available are universal, ie, Crohn is the same here than anywhere else in the world. I insist if the environment is not a drug, it 's the same with the chemo. Chemo is the same here as in Sebastopol, but the setting is human. Well attended ... well comforted's what you need to be with people who understand their worst moments, that's the hard part . You go to the emergency room with an acute flare of Crohn, you get to the ringside doctor and gives exactly the same thing, you go into digestive and digestive tomorrow will one see you. *"A seventh Crohn income, as usual ... Dacortín , just make it a TAC ..."* Do you feel well cared for? Because you are doing everything possible, putting in the protocol, but no one has sat ten minutes with that patient and see what happens, to explain what has ...

**And according to your words the person who has to do that is the general practitioner?**

In the hospital ... not in the hospital a digestive health. But follow-up if it has to make a general practitioner. But when you walk into a hospital to you send to internal medicine and internal doctors here know there is. And the doctors here spend time with you in the room. If what I think the great thing about private medicine is more individualized attention, in my opinion. And everything else is equal, treatment is the same, but call it that the public is more dehumanized, let's level nursing and auxiliary luxury, but the medical partner, overwork can not devote maybe long as he wanted.

**Interview E**

**Primary-community doctor**

**When you get to a patient already diagnosed with EC come How do vivencian?**

Mmmm good, you have to have to be closely monitored patient is detected and the patient needs continued support, explain that anything that quickly see that it is preferable not heavy small otherwise. But they are usually very well directed from the consultation of digestive and we only intervene promptly in cases to consult us for medication. Then you know that the medication makes the specialist but those who do the continuity we, thus, do not lose contact with the patient. But we go…

**What do you think is what most impacts of the disease?**

The strangeness and curiosity, that is the problem they have is that they want to go so fast, they want to learn everything eh? And they are always looking for complications, they will always putting in much worse situations to which they, ie, have a fear a bit exaggerated at first because the same curiosity makes them more research account, ie, want more information on a principle which I believe are able to assimilate. Think from above (referring to the hospital) he says it is a serious disease that can have serious complications and come with such an excess of information for what they have said and curiosity you have, you already know that now, you say one thing and you'll automatically check the Internet, which is true,thus are often with late complications and already put in the worst, then as I said, if there is such a degree of rejection, because it is an unknown disease and it is a disease unknown, we are faced with something that nobody has them explicao , who have not heard of it and I'm telling you is this

**So you think you have too much information?**

Correct, and also that process in excess, usually come well diagnósticaos , Well orientaos but come as you have said, and good "I do not know if you will I be able to help because this fits very big" want still more, because at first think they're weirdos until and they get used and they are realizing that it is not uncommon that if you search is that even within your environment there are people who have and who live and can live perfectly. But first visits come very teach and thinking that the worst is going to happen to them, they will do them resections ... and I say that not having that evolve as well, but they do have that thought poor prognosis, along with you (family doctor) I can not tell because this is an important disease. Then we were in the first phase in the background to say, canceled.Because it is a first hospital diagnosis, with some specialized treatments and also because from the start out with "anything that happens, you come to the hospital, you do not see your doctor" eh? he is already saying anything here.

Also by medications, because there are some that are unique to non-specialists and you can prescribe, or can not put in the health center, and then you stay out a bit like ...

**You do not make them any recipe?**

Look look, the same system has favored you can not track them from primary, because before you when they came to take the recipes, doing them follow ... but now when they come you what you do is that if they need any medication and you have said they have to take it for six months, you will do the treatment for six months and up to six months from now will not see again, anything also have your phone reference, then come here and tell me "is that I llamao my doctor and he told me to do this to me "but nothing else, and consultation have made with them. Also do not ever change them any medication or anything, because they only do it if the hospital ... for example I remember a case that I did not notice and prescribe a medication in suppository once said "me needs to change, because it has to be foam "... or for example diet, because not as you know there is a type of food that is found to affect more or less to the disease, but of course, when you say he has to be your own doctor about this, the patient understands it as you do not know anything about the disease that has ...

**So you know how to live with the disease?**

No, I tell you since we have the Abucasis , we almost lost the inaccessibility we had them ... sometimes even I have heard that they have to go to control and I propose to make analytics, they tell me no, that although with analytic made, there is the re-make. We dare to say that it has a sectarian tinge, let a closed shop and it is very difficult to enter as top doctors are bound, if you suspect that you may have Crohn you have to refer the specialist ... and you lose it . Well if as a result of Crohn's person has some social problems such as depression or anxiety, it does come to see, while I was dealing with the anxiety-depressive disorder could track them when they were resolved no longer knew more of them

**And now that you're thinking What do you think of this situation?**

Let neither hot nor cold, stay a little outside, they are somehow hidden patients ... and as far no one has told us that destapemos because we do not touch anything. But do not think that it is only with these patients, it also happens with diabetic children, pregnant women presenting with diabetes ... Let no relationship between primary and hospital, but this is not now, this is for more than 30 years ... but of course if to me it occurs to me to change any medication, however small and the patient suffers the slightest change, I've cargao ... I mean, my role is to tell "this treatment as you wear it for life "I can not intervene anymore.

**Where, in your opinion should be supported?**

I think from self-help groups or what is now bears that entered the expert patient program, leaving primary ... that would be an idea, but is that this group of patients have them forgotten. It is that right now, we can not do anything ... see if I think of my training on the EC I have to go up to the race ! Other diseases we have made training courses, such as thyroid went from being exclusive to specialists to take him ourselves, but is that this ... nothing is the great unknown, the conclusion is that they have a complete and utter dependence specialist

**So you do not consider part of your support network**

No, not at all, but not by me but by entering a kind of ritual and do not go there, but I imagine this will change changed as diabetes, thyroid and is now happening with cardiac patients

**Interview F**

**Primary-community nurse**

**How is your working relationship with people with EC?**

When they come to the center and the hospital diagnosed. They are affected by the disease, but other than that, they also expect you-involved you, I mean, for anything they tell you "Hey, remember that I have Crohn Does this I'm taking it right ?, you-involved in preventing not to aggravate his illness, yes, they are always aware of that. Not as much as I know I'm sick Crohn but do not go to hurt me, I do not agravéis my circumstances, you do not do anything that could hurt me, yes.

**You're telling me what's certain distrust in yourself?**

I know it is not you talk about the hospital, but the possibility that we may be iatrogenic, for patients I know of Crohn's obsession is that you're wrong, you give them a medication that is wrong, that ... stuff. What can you do wrong rather than what we can do well. Anyway also depends largely on the stage and severity of the disease, is not the same one who can do the activities of daily living, which has been no need for any operation ... Let's almost like are made defensive a little of us, we like we were iatrogenic, as if they knew more about the disease than us, there is a situation here in which the patient seems to want to take responsibility for his illness, but in a very solitary way, from the first time.They think that professionals do not have the knowledge, because it is a rare disease ... and then I tell you that almost examine you. It's a story you have to tell them "do not worry, I do not know, I'll ask the doctor and if you do not know you're going to say and that if a medication contraindications I'll be aware "are distrustful, they think things will they know all of the disease do not know it? And then since it has no known origin also they have an interest in controlling not want to pass the doctor full control, because as you know that the origin is unknown and that treatment is there ... they are already like waiting ... and very afraid to hurt them.It's a story you have to tell them "do not worry, I do not know, I'll ask the doctor and if you do not know you're going to say and that if a medication contraindications I'll be aware "are distrustful, they think things will they know all of the disease do not know it? And then since it has no known origin also they have an interest in controlling not want to pass the doctor full control, because as you know that the origin is unknown and that treatment is there ... they are already like waiting ... and very afraid to hurt them.It's a story you have to tell them "do not worry, I do not know, I'll ask the doctor and if you do not know you're going to say and that if a medication contraindications I'll be aware "are distrustful, they think things will they know all of the disease do not know it? And then since it has no known origin also they have an interest in controlling not want to pass the doctor full control, because as you know that the origin is unknown and that treatment is there ... they are already like waiting ... and very afraid to hurt them.they think will you know all about the disease do not know it? And then since it has no known origin also they have an interest in controlling not want to pass the doctor full control, because as you know that the origin is unknown and that treatment is there ... they are already like waiting ... and very afraid to hurt them.they think will you know all about the disease do not know it? And then since it has no known origin also they have an interest in controlling not want to pass the doctor full control, because as you know that the origin is unknown and that treatment is there ... they are already like waiting ... and very afraid to hurt them.

**Do they have more tendency to "trust" somehow more nurses hospital in you?**

Yes, somehow yes, because they have the thought that my doctor is the hospital, and the nurse who knows about my illness is the hospital ... it's true, now that I hear tell I realize that it is always all some referred to them. Look even when they come that have operated as they come, if they come with all the recommendations of cures nurse specialist and you can not change anything, and of course here's the least and you have to settle, and that gives them confidence, let If you hit him and it solves the problem for which he has come back to see it a little bonding, but if not, link the hospital and you can not approach them. And I imagine that the doctor will be the same for the time they spend with them in the hospital. It is the diseases that are linked to hospital,Note that I had not thought about that ... even I call the nurse at the hospital, and asked about the patient, it is he who pierces me its link with the other nurse at the hospital, so I am a mere bridge. Nor will we give us much choice to go, we can not help with diet or with exercise, nor smoking cessation ... Because in the hospital and have their reference nurse if you have any doubts get in touch with her.nor smoking cessation ... Because in the hospital and have their reference nurse if you have any doubts get in touch with her.nor smoking cessation ... Because in the hospital and have their reference nurse if you have any doubts get in touch with her.

However the system also provided us training courses on Crohn or basic care about ... nothing, nothing. You know, let it just as we have been formed in diabetes, hypertension ... of Crohn nothing and neither is the interest to do so. So I understand that currently have no interest that we treat them here. Then as anyone to take interest in that we are in, because we are out ...

**Do not you consider yourself part of your support network?**

Not the fundamental support of them is the hospital, if I ever have recommended them something to a family, we always say "good mom, I called the hospital and asked ..." They're always like on offense, we can botch and we can make you one iatrogenia that will complicate the disease, that is the feeling I have as a professional, then you put yourself available when asking the question, whether directly know the answer and if not you know you say "I hope that I get in touch with his doctor or the hospital ..." and although they answer, then you say "anyway and I call I am to ..." are patients who can least enter. Let the feeling is that we mess up, they are also patients who carry all very controlled, testing, analytical,medication ... they have recorded them to fire in the hospital who have to control very well because if they are not going to complicate then they control well or think they are doing well, then sometimes they are exhausted, but of course ... We do not enter. We have not done anything to enter, no protocol to enter ... But neither are patients who blow off steam, seeking to ask you things, have the mindset that the health center nurse does not know what to do. It's like the fellow hospital has not delegated enough for patients to trust the health of the health center, that happened before diabetes ... and at the end you see we who took control of diabetes.then, they control well or think they are doing well, then sometimes are exhausted, but of course ... we do not enter. We have not done anything to enter, no protocol to enter ... But neither are patients who blow off steam, seeking to ask you things, have the mindset that the health center nurse does not know what to do. It's like the fellow hospital has not delegated enough for patients to trust the health of the health center, that happened before diabetes ... and at the end you see we who took control of diabetes.then, they control well or think they are doing well, then sometimes are exhausted, but of course ... we do not enter. We have not done anything to enter, no protocol to enter ... But neither are patients who blow off steam, seeking to ask you things, have the mindset that the health center nurse does not know what to do. It's like the fellow hospital has not delegated enough for patients to trust the health of the health center, that happened before diabetes ... and at the end you see we who took control of diabetes.no protocol to enter ... But patients are not let off steam, seeking to ask you things, have the mindset that the health center nurse does not know what to do. It's like the fellow hospital has not delegated enough for patients to trust the health of the health center, that happened before diabetes ... and at the end you see we who took control of diabetes.no protocol to enter ... But patients are not let off steam, seeking to ask you things, have the mindset that the health center nurse does not know what to do. It's like the fellow hospital has not delegated enough for patients to trust the health of the health center, that happened before diabetes ... and at the end you see we who took control of diabetes.this happened before with diabetes ... and at the end you see we who took control of diabetes.this happened before with diabetes ... and at the end you see we who took control of diabetes.

He is also young people, let most cases, so they are useful for life, useful for work and do not have that depend on anyone ... are not whiners have a fighting spirit and do not want to depend on anyone, the trend generally they want to get on with life ... so you never ask me, I know nothing of them, have never told me anything intimate. Only when they have to be analytical control, you talk a little with them ...

**Within your prevention campaigns Do you include the Crohn?**

No, because nobody told me, they are for diabetes, snuff, bullying ... and now from January enters the campaign to prevent colon cancer, but starts as early as 48 years, so never cases will be detected in a primary moment because the EC makes debut at younger ages, ie prevention do nothing. Notice that I would never, crossed his mind to prevention or detection campaigns on EC institutes ... I think it would take some sort of consultation of young people. Ultimately it is not prepared primary to treat these cases.

**Who do you think should be professionals to support people suffering EC?**

Man, as chronic should be a multidisciplinary team and be inter-related to the health center and hospital, depending on how it is valued ... well from the point of view of the patient, now I do not know, but from the point of view as a nurse should logically be a multidisciplinary team ... and of course counting on the family. Let that should be in the portfolio of services, but the wallet brand the company as the company did not tell us anything ... it is hidden, and also when you have an initiative ... because no can develop initiatives must come from the address can not from a health center, are worthless, you have to be more multicenter , no ? It is very difficult ... and the patient should ask where they feel safer, because of course ... your opinion is the most important, but of course it is conditional and will never feel comfortable with us. But this is true in all cronicidades, with diabetes, with Sintron ... all of them have evolved, there have been some power groups, which are those that determine how you have to organize health, c'mon how services are organized and who have to take care of the different cronicidades. Come on we're not used to working together, but now or 30 years, we who care focused on the patient anything, everything depends on the power groups, if they decide they want a disease just for them, because others can not enter

**Interview G**

**Secondary-hospital nurse**

**What do you think is what most impacts a person who was just diagnosed EC?**

I think at first what matters most to them is to stop symptoms, so first time. Because normally when they have gone has been so for some exaggerated diarrhea or have begun to bleed or were long losing weight ... and then at first, it seems to me, what worries them is to control symptoms. And "hindsight" and that's when they drop the yoke of chronic illness and that's when and begin to assimilate and to ask *"but then this is not going to cure me? ¿ And I I have to live with this forever? "* and I do not think at first assimilate and understand the information they are being given ... They are very young people 18 or 20 years and then there is always the mother figure, above all, that has been a bit that goes with the baton , typical phrase mother *"you do not worry that nothing happens, if it is controlled ..."*and sometimes view them face and thinking *"if it is , you tell me not to worry but now I'll be here fastidiao but ... now I'll be able to play football? Will I be like that for a lifetime? "* and sometimes they ask you all, for example when they have to get cortisone enemas or so they ask if it is for every day of your life, and then we take advantage to go slowly giving information. They ask many things but mostly on plan *"and will be able to do this? ¿ I will be able to do that? ".* I think when you tell them the disease is lifelong associate it with any other disease because in plan, I dunno, like hypertension can not eat salt, in diabetes and can not eat sugar or I have to put insulin, because when you tell them that the disease is forever, what worries him most is how much they will affect their daily lives and their activities that make daily, rejoin and lead a normal life. Then the consequences that may get them or treatment you have to keep carrying ...

**Before dealing with the disease and the limitations they ask?**

Normally the patient does not usually ask, often ask the companion, because it gives me the feeling that at that time the patient in shock and not very lucid mind at that time raised many questions ... Let's you overwhelmed by the situation. You think the patient has reached emergency and maybe has come to the emergency room thinking it was a gastroenteritis of these or salmonella ... and suddenly you have a colonoscopy, which has been a super nasty test, which is also a trauma certain way, and on top when they have the result they have been told that what you have is a lifelong disease and over're told you can not explain why it happens. Then start thinking, if a young person, more or less healthy habits,and then you ask why it happens to you that? And suddenly you hang the poster of chronically ill for the rest. And I think at that time the information you give him, slips, because they are unable to assimilate. Perhaps most occasions they paint them more black than it really is, always they put the worst of the worst. And the person who demand information and more is in control of the situation is usually the one next to the patient, which is what is as colder, more whole and is what is there to gather information to order a little life until the other person is able to take a little charge him. And in the case of young people, they do not paint anything, are asking their parents, take you out so you do not hear *"and then my son will he have to wear diapers?"* . Then afterwards when you already are patients who know the disease, I prefer to ask them, and keep to them really, removing things on prescription are Sota, knight and king other things, dietary measures or things like that, I I prefer to follow them because they really are the ones who manage their disease and those who control it. In addition I see absurd they go to class to something that I do not live. I have been with patients who have debuted and since I have not seen, I would assume that when you have an outbreak or have had very little or when they have had has been something so slight that has been solved in door urgently required and has no income and then have been other, do not ask me why,but two months have again and enter the six months have returned to enter and then have to operate. Let that since he made his debut does not leave the hospital and now parenteral nutrition and now do not know what ... then these patients because you see things such as a young little girl who had recently was with parenteral nutrition and notes in the room and I said *"But girl you're bad, that parenteral nutrition is not compatible with notes, rests, takes strength, Recupérate"*and say *"is that next month I have exams"* and I thought that maybe within a month was still entered because I was with parenteral nutrition, not tolerate anything, you have digestive rest and still do not know if you're going into surgery and they'll cut a piece of intestine ... This girl She wore it as assimilated, let it understood that it was as a stumbling block would be about fifteen days in the hospital, they were going to do anything, but a fortnight she would incorporate into her life and she had to to face and had to keep his things everyday. Then yes they usually integrated into curriculum work plan, because you walk into the room and the look with the laptop. And in that sense still you see that a lot with his life, that outbreaks are not a complete break to say *"I fell, I broke everything and now I have to start over again , "*we will take it as a break of one week to fifteen days, but once you leave the hospital is continuing its life.

**So you do not live outbreaks as limitations in their daily lives?**

Well , look, in the hospital I have not seen a lot, but I also do camps and handed me a case with a child who came from camp because his mother would not let him because he had Crohn, if had outbreak ... and end the last year we got her to come, calling his mother every day, let's that her mother thought the child could not swim in the pool if he was not alone, I could not go walking, I had to eat very special things ... then the boy was like a bubble you know? The child had been limited, but for her mother, if I had let the disease is bone glass ... let you saw the list of restrictions that took the child and we was all no ...

**Overprotection?**

Yes, yes of course, but also an overprotective based not know what, we do not know what beliefs or what knowledge of the disease ... And this overprotection's also see in young children who are admitted to plant. I tell you that maternal / paternal figure is the one that will set the pace and giving you directions to patients what they can or can not do. Let the star is directly account for parents *"because I am the mother and know what is good for my child, or because it is my poor son and I will not bear all this"* and also there we noticed a lot in young patients is the mother who picks up the baton and is telling you what you eat, what not eat, which restricts visits ... At first during the first years of diagnosis, you see come to the mother and you know who's coming and what's coming, and they are very demanding companions, who want to dominate 100% the situation, they know what is right for your child and is your criteria but then since when they become older, they change the figure of the mother of the bride or by the couple, which is what takes a little support, but as a general rule once the mother disappears, takes much weight the patient's will and knowledge of the patient. Not usually convert the couple in another mother, but is the person who is already known, and is managed,and it controls the situation, the couple is aware, is involved and supports but is not that radical domain of mothers

**What professionals seek support?**

Well ... see, there are nurses on the ball, which whenever they have any questions or something, I tell you, and tell you that they forgot to ask your doctor. To us they ask us enough, then, for example, auxiliary, as well as spend more time in the room, because we is that we entered the room and almost we do our work and is already and ancillary come, know a little to the mother, they are also mothers all have children, empathize a little more, your child speak as they are released, it's like that favors a little more informal atmosphere, pass them a series of questions that you dare not hacérselas to physician and the nurse comes in and out because neither the questions. But of course,asked the assistant and maybe the auxiliary has no idea and then as you go and solve the doubt, of course. But as a general rule, I say I think the doctor, the doctor receive, receive, receive ... assimilate part, because then there are many things that tell them they do not understand and do not ask for fear or shame that paraos stay and is that really the nutritionist for example, the dietitian if you are released again in plan dietary and such recommendations itself that usually have very good relationship, because eating is one of the most important things in life , then dietary restrictions is one of the worst yokes that have this disease. And the fact arrives dietitianPhysician receive, receive, receive ... assimilate part, because then there are many things that tell them they do not understand and do not ask for fear or shame that paraos stay and is that really the nutritionist for example with the dietitian if they are released again in plan dietary and such recommendations itself that usually have very good relationship, because eating is one of the most important things in this life, then dietary restrictions is one of the worst yokes He has this disease. And the fact arrives dietitianPhysician receive, receive, receive ... assimilate part, because then there are many things that tell them they do not understand and do not ask for fear or shame that paraos stay and is that really the nutritionist for example with the dietitian if they are released again in plan dietary and such recommendations itself that usually have very good relationship, because eating is one of the most important things in this life, then dietary restrictions is one of the worst yokes He has this disease. And the fact arrives dietitianwith the dietitian if you are released again in plan dietary and such recommendations itself that usually have very good relationship, because eating is one of the most important things in this life, then dietary restrictions is one of the worst yokes that have this disease. And the fact arrives dietitianwith the dietitian if you are released again in plan dietary and such recommendations itself that usually have very good relationship, because eating is one of the most important things in this life, then dietary restrictions is one of the worst yokes that have this disease. And the fact arrives dietitian and soften them a little all those fears of principle, *"oysters, I will not be able to go to the Mexican never in life , "*to tell you something, and the best dietitian comes and teaches you how to cook food, what you can feel bad ... But in the case of nursing can not do more because we really do not have the time necessary to require that patient, then the assistants have a deal like more casual ... let nurses do not tell us things they do in the day a day, well you know when they return. When you re - enters with outbreak or a complication, as you know him before then maybe you have more, but because they are already better known patients ... but of course, you always stay in a superficial plane ...

**For you Are all support needs within the hospital?**

No, not at all. But often I think the support you seek out, self - help groups or associations plan you know? As it is easier to ask and share with people who are like you, maybe get help from a professional, I do not know what seems to them like superprofesionales and you do not know anything, so often rely more on partnerships chronic rather than seek professional help. Yes I tell you in hospital for example, would be nice as in hospitals there are nurses who are experts in ostomy okay? They are there to support 100% in a patient ostomy and indeed are the cornerstone with which the patient starts walking, once you drop the world over for having the ostomy and he does not know what to do, I also consider that there ... like a nurse who could lend a hand with diabetes, for example, because we health education can do is *"teach you to make the Dextro and teach you to get insulin "* and ball point. When you want to do something more, but why you do not want because nobody tells you, because you get into the room when you have a gap and explain what foot ... because it should be that when someone comes with a debut we planteáramos the goal that when the patient out of here, have a dietary management and exit with all the result doubts ... and that for example if it would be really interesting to have an expert who was the reference person and who was the person who could would solve all your doubts so that when the patient leaves the street did not say *"my mother now what I do with it if I have a medical report alone?"*

**Who would be that professional?**

Ah! I have to advocate for nursing, we have a nurse cures that is a benchmark and we work great, nurse ostomy elsewhere that works great in that sense I vote for the nurse. But of course, it has to be a nurse who is working a little discharged, in order to have more time to devote to it. Because if you have to do in your work, I understand that is not the right way or you're in the mood to do well, nor will transmit to that person availability you want to convey good because in plan *"good for thou when any doubt you can think of me as you say and I can come and I miss a hand or speak "*. For example, the need information, because they ask a more logical others with less logic, you will answer them as best they can and based on what you remember, sometimes you have to say you do not know and that you have to report, but Driving the situation not to say "ask me anything you want to know I'll help you" never ask for sex okay? It is a taboo subject and super-Cortao, maybe the older people themselves that make some reference, but let young people or chance. But I think they are lacking information, on the other hand what is the daily management and symptom control, alarm signals, the ... the what you expect to catch you by surprise, and what you can do with these signals or those things that are going to happen, how will affect, how it will be your life,throw some myths on the ground and reinforce him the things that really know those attitudes and motivation, overcoming ... I think support would go in that direction, not only of information but on the other hand that little push of encouragement.

**Interview H**

**Expert nurse in digestive**

**How do you get people with CD who have an ostomy ?**

Normally they reach three - way primary. One is when they reach the primary doctor prescribed them and need devices, I have agreed with my medical center that at least advise them to come to see me, to talk, answer questions and others. Which I receive, if it is true they have some information because they come from the query of San Juan, that there is consultation osteoterapia , but for example, which come from the hospital in Alicante, which is the reference and have consultation and then come via derivatives, so surgeons, to advise them , make them follow - up, complications, questions ...

But my place is a nurse primary, I of ostomy I do it because I like it , or the management knows that I do, because the argument was "if I have a query chronic, and ostomy is a chronic patient because I will not deny assistance "and also do training for other colleagues to follow others.

**How do they live their illness?**

As for the disease, at first I think it's a little ignorance, until it is having complications and is having buds and know what their acute process and what is its most stable process that has maintained illness until no they go through those processes I think are unaware that the disease is. With the theme of ostomy , I think they have a higher impact when they are diagnosed with the disease, because it is very visual and in that first glance, you get idea that impact will this have for my life, as to image, social level, on the role that came to play and not now. However, with the EC, I do not think the first point to be aware that they will not discover.

**Do you feel you have enough information about their illness?**

Do not have enough information, nor as to their underlying disease or as to the ostomy . While less information than the underlying disease, do not have nutrition information, some care ostomy , but also very little. I have an advantage in that while they are in the hospital process, they have a short stay, management with ostomy need to know fast in three, four days ... but do not see to do when a complication arises, what tools I have available to treat them, how I can prevent them ... then that is not enough time to see him in the hospital and yes I have time to treat primary. Like I have time to talk about sexuality, work role, problems of disability ... I do not know things I'm doing and all that they learn if they have that follow, people have no choice this track, certainly not has.

So is the support level networks. As for the formal network, they have no support in fact I tell you ... for example in the Hospital of Alicante that brings more than 200 ostomy annual and do not have any follow-up high ... you have no recourse, people have the option to reach a site that make them track as they do in the health center, well they have some appeal, but the remainder is zero, the only recourse they have is informal, people who have supported is the only one, but people who do not have that resource primary ... I have many patients and is one of the key points from the center health and home care is, there are many patients who can not move even to the point of basic care, which is the health center ... then all of that at the hospital level is lost and equal support. So they rely more on the family, because we have mounted in the health system, it is the family.But this support is only enough to cope but not to take it the right way. Yes it is true that without such support certainly could not pull it off or not more or less adequately, but not enough. For that support, in turn, need another support and how that advice, what would be the most appropriate way, to bring forward such support.

Optimal resource, in my view would be a nutritionist or a nurse with advanced nutrition training would be necessary to achieve stable maintenance. And in general multidisciplinary team from the health center. I have contacted the hospital, because I've been working there about 10 years and then who is going to come. But I do not get any patient derived from the hospital, a patient indeed has come i am continuing with regular hospital follow - up, there has been conflict, the fact of taking from primary care. They are told that the health center do not have to go because the ostomy is've done in the hospital, even I have received calls telling me that I can help and do not have to send them to hospital.

**Interview I**

**Nurse Specialist**. **Day hospital**

**Where do you desempeñabas your work?**

I was in the hospital day and we tried sick of all kinds, from coming to get intravenous iron because iron deficiency anemia had and they were fine, they had no outbreak, even people was wrong. I was there for three and a half years, so I've met almost all cases ... In fact now I keep in touch with many, even through social networks

**What do you think is what most impacts a person when you say it has the EC?**

They all made me the same question when ... well first they when they diagnosed, we had ... but hey, that it was my initiative. They told me that they had diagnosed a new case of EC and the first thing he did was go to the room to visit them , because of course, first diagnosed in hospitals. They are first blocked and then not really know what is the disease and think almost everyone that is what they have eaten you know ?, everyone tells you "will have eaten bad, I did not feed well ..." and has nothing to do with it , c'mon that's the first thing you have to say, that has nothing to do with the past who have had or what they have eaten ... the fear now is whether they had done something so that this disease would have the face you know? *"And why it happened to me this?"* nor can it explain why it is not known ... the first thing you ask. And then very lost, very lost ... are best for them it was to explain who I was, who was her nurse reference, give them the phone number to call me any questions. This is not designed to function nurse day, I did because when I arrived at the hospital day were very lost. When you are sitting there getting treatment, detect they have a lot of questions for them are a world ... and it's really silly, I did it because I was a nurse, and I liked digestive, the first contract I worked as I did in digestive and then I majored lot in digestive and I do not like to be a nurse to get into a room to put medication and over,c'mon that I have never done

**What do you mean you've specialized? How did you do it?**

See, at first, had a scholarship at the hospital, they wanted to put a person that will handle the day hospital, because they wanted the most controlled patients, because every time they were putting more biological treatments and wanted someone who was Hence, if many patients came to the emergency room ... because many patients could be treated at home ... so they decided to apply for a scholarship to a laboratory for a nurse and I proposed it to me. They are also getting treatment over an hour and you're there with them, one tells you one thing, another tells you otherwise, because most are young and they do not know why when in the doctor gives them trouble ask certain things, I do not know ... and if course,You give them a phone and tell them "for what you need me" even after many who were very unwell they were always there to give them got my personal number. Yes because there were situations like "I'm so what do I do? I go to the emergency room, I hope to Monday "and then you have the other party, even if you're a nurse had medical support. Because you can have a lot initiative but the doctor will have to support. And you have to have ordained, I was in digestive and then I recommended several guides to know them and then of course is three years and you learn a lot. Then of course the same laboratories master offered me that I have a masters in inflammatory disease ... But that's because you love your job and you notice that there's a big gap. I even pass the first visits of consultations, I made them anamnesis and was studying them colonoscopy. We're going to have to work together with doctors and that's what I have left to me.

**What do you mean by empty?**

The absence of a reference person in the hospital ... right now I got laid off and there is another girl, and so I have told the other girl has nothing to do with what I was doing ... I just ... to my me things have patients and I wear black ... but of course I have to look that this was scholarship and finished ...

**Do you consider that realizabas support or adequate follow-up?**

I think emotional support first, because I do not know ... I have not studied psychology, but you know when you meet a person far you can go or even what that person can offer you know what I mean? When a person starts to tell you "Look, I'd like to have babies and what this treatment can have children?" And you begin to tell personal things because you know you can go to support them more. And there were others that a barrier is put and not let you in, we do not know if that was the best way or the hard way, because I'm not a psychologist ... and depending on what you demanded me as I was answering. Some worried maybe physical, for example ostomy , because they had ostomy very young, as if they had relationships, what they could do if they wanted to have sex, if there was a plug that could be used to cover that ... we did not know me much, but I was going informing and training on the fly. Others were psychologically very tocaos, spent the day at home because getting out and give them a squeeze for them was world, many had, we almost all "me is that I go out and walk into a room and the first thing I do is to detect the toilet, and once they had located the toilet relax me, but until we had located the toilet ... it was like ... "traveling, some of them I said" Well, no I can get on a bus for the trip lasts eight hours and I if we have to stop ... I've had that for a bus,I told the driver to stop or shit or above "and of course this is so, for that more than anything ...

**You mean limitations?**

Limitations? That they want ... such as traveling, I told them that they could travel well, they had to lead a normal life that had to be programmed, for example to get you going traveling, then Take some pills ... we gave them recommendations . To me have come to ask me "Is it time to get pregnant?", The doctor will not ask that. But maybe asking, because I listen to them, maybe in the day hospital and I were asking questions ... because the great thing about it is that if there were four treatments, four treatments were sitting and I was trying to do them the same age, so you always coincide the same people, and everyone had things their illness and including recommendations were made and claimed that they were the same age, because it posed the same problems, such as leave, sex ...

**In your opinion the right professional to support / carry out monitoring of these patients who would it be?**

Nursing, no doubt. Because we have several branches: one medication level, if you have an outbreak, we are the guide doctor because we can contact directly with him and two because it is so important that treatment works well for them, as the nurse puts it be for them confidence to talk to them. Because most go to the hospital a lot, because it is a disease that many controls every three months, every three months ... then I think I do that if Drifts many professionals, in fact, I tell you why ... they, did a vaccination campaign because I was it was better to inoculate because many were immunosuppressed and decided that in fact the study is that if we do not we derived a preventive or not we accompanied them to get vaccinated, they did not go. Come if you just come to the day hospital,in fact I remember it was the vaccination campaign influenza and recommended to all, and they were saying "come on, you see vaccinated" they said "good and go ..." and then I called low and sent directly, and so everyone get vaccinated. If you leave them at the end for them ... is a mess, because they go way to the hospital and are very lost ... For example, in Barcelona Vall d'Hebron there are two nurses who are responsible for this, are two of inflammatory and one of colon cancer. And that was the idea that we had in Alicante, what happens is that I'm already gone and the girl is no time does not want to know anything, we mount a drive like Barcelona because so desaturated consulting doctors because they are up with work, because they see 25 patients spend two hours ... and that we do not get to do because there was no physical site, even we were looking for an empty query each day to see if you could do. The digestive told me , "it is that we take off only queries patients who come for controlling the start of Imurel Which they are, for three months, a week, a month and three months (and did so) "is that you have to think that the patient is involved: removed analytical, one day, job loss, other day you go see the analytical ... and so many more. In the end what happens? If I am not going well, because that is so. When you come you see are fatal, coming with a pain that die and the analytical half a year ago that have not done. Let are typical patients who endure long in coming, because they think "and I will," people have come to me with pain, twisted and have sent straight to the emergency room. And I said "get me something that I have to go" ... go and was occluded, no, this ... and let that patient with an ileostomy was because he had no columbus,or whether that person knew more than the disease did not know anyone, but still endure. Do not want to enter, and tell you "do not want to lose the job" ... We then are extremists, you called to ask if they could eat an omelette, but hey, after longer depends on the fear they had to enter. Because almost always those who were worse off were the worst patients, because they knew what it was, that if they came entered. Those who did ileus, and you came because they were taking corticosteroids, on their own, and did not improve

**Is there a good medication management?**

There is a lot of freedom in many cases when taking medication, but also depends on the doctor, let the doctor should know who can give you freedom and who is not, and that's the problem, as the doctor does not know patients, because they are not able to know whom you have to explain without restrictions and who you have to explain like a child ... people have been very badly and has taken a treatment and is fine and so adherence to treatment is very good and there are people who have found very badly and has begun treatment and has gotten better and thought "I'll treat me to see what happens" and then they have fallen again.

**Who do you think you look for support?**

I guess in the family, but also for the family is like something very unfamiliar and sometimes gives advice that are not good. When I took many were very lost indeed came many with companions, because they were always accompanied but I remember one case, a boy of 40, these people see that you are diagnosed with a disease and is acobardao completely, face that of asustao you did not ask anything because I was asustao and always asked the woman and put a treatment at the day hospital and the woman asked me but he did not ... and once an analytical took off, I said, "Look analytical" because yes, for me the analytics is the patient, I have no reason to leave analytic here and say "everything is fine", he explained everything ... and it happened that came one day only and started talking to him and I never came to women, mean that is first you you feel like a professional, and then just listen a little ... I think the family has them as a little sorry, I know I get that feeling, like "how young and sick for the whole life "say that phrase a lot of times. I think the EC for society is the great unknown, and this is what makes the family more over,because for example you tell you that one of your family has cancer and family know how to behave, we are going to have to be on top, but when you tell them they have the EC and that's for life, it's like, not caught as a chronic disease such as diabetes ... then I think it is, that family unite around them, then they will query to also learn about the disease but they do not know what will ... support the family is going with them to consultations, we I see them are like companions, let that if they say something in the query are not alone ... for me, first the real support is given in self-help groups, let them see that not everyone is the same ... I told them the first thing was not to get involved in internet and be with more people who have the diseaseand be with more people who have the diseaseand be with more people who have the diseaseand be with more people who have the diseaseand be with more people who have the diseaseand be with more people who have the diseaseand be with more people who have the diseaseand be with more people who have the diseaseand be with more people who have the diseaseWe're going to have to be on top, but when you tell them they have the EC and that's for life, it's like, do not catch as a chronic disease such as diabetes ... then I think it is, that family join around them, then they will query to also learn about the disease but they do not know what will ... the family support is to go with them to consultations, we I see them are like companions, let that if they say something in the query are not alone ... for me, first the real support is given in self-help groups, we see that not everyone is equal ... the first thing I told them was not to get involved in internet and be more people who have the diseaseWe're going to have to be on top, but when you tell them they have the EC and that's for life, it's like, do not catch as a chronic disease such as diabetes ... then I think it is, that family join around them, then they will query to also learn about the disease but they do not know what will ... the family support is to go with them to consultations, we I see them are like companions, let that if they say something in the query are not alone ... for me, first the real support is given in self-help groups, we see that not everyone is equal ... the first thing I told them was not to get involved in internet and be more people who have the diseasenot caught as a chronic disease such as diabetes ... then I think it is, that family unite around them, then they will query to also learn about the disease but they do not know what will ... support the family is going with them to consultations, we I see them are like companions, let that if they say something in the query are not alone ... for me, first the real support is given in self-help groups, let them see that not everyone is the same ... I told them the first thing was not to get involved in internet and be with more people who have the diseasenot caught as a chronic disease such as diabetes ... then I think it is, that family unite around them, then they will query to also learn about the disease but they do not know what will ... support the family is going with them to consultations, we I see them are like companions, let that if they say something in the query are not alone ... for me, first the real support is given in self-help groups, let them see that not everyone is the same ... I told them the first thing was not to get involved in internet and be with more people who have the diseasethen go to the office to also learn about the disease but they do not know what will ... The family support is to go with them to consultations, we I see them are like companions, let that if they say something in the query they are not alone ... for me, first the real support is given in self-help groups, we see that not everyone is equal ... the first thing I told them was that they would not get on the internet, and be with more people who has the diseasethen go to the office to also learn about the disease but they do not know what will ... The family support is to go with them to consultations, we I see them are like companions, let that if they say something in the query they are not alone ... for me, first the real support is given in self-help groups, we see that not everyone is equal ... the first thing I told them was that they would not get on the internet, and be with more people who has the disease It is good, including talk much and then have to see a professional to guide them, because well ... and this can be done perfectly nursing because many ... maybe a consultation nursing itself to say "come and talk a short time "that no, that would not work, because they are so, if they have something necessary to go not go, we would go four because think" if I feel good that I'm going, "let it as did I, I managed them analytical, testing and took advantage of the moments of waiting for results to put them together and they could talk ... you have to think that the person you have as a patient is like a family ... and now they have made a fatal thing in the hospital, because they have unified the day hospital,and EC patients are getting treatment and next have a person with a tumor that is fatal, and this should not be mixed ... because they are psychologically not so strong.

**What coping strategies made to maintain normal that you were talking about?**

Many of them trying, the disease is much to know and learn how their disease, because it is a very individual disease and they ask a lot. There are many who want information but do not know where to take her, others put the mask and do not want to know anything. Yes it is true you can now give you information and tomorrow out something else and having to give it another different information, but unfortunately in this disease will progress very slowly ... or for example I explained to them what constituted the treatments and possible side effects that could have ... I am of the opinion that the best to lead a chronic disease, get to know your disease, which teach you how it works and what cosiste.

**Interview J**

**Nutritionist**

**How do people live with their disease EC?**

When they arrive at the consultation, are very concerned about how to change the diet because they do not know how you will react your gut and are very afraid to return to a crisis diarrhea and such, then gives them a lot of fear put vegetables, it gives them very afraid to do any changes involving them return to a crisis, because usually when they come to consultations been in a period in which, maybe the Crohn was not the target, because they had more controlled with mediation and such, because it best to was to lose weight or because they have health problems, because they were going to operate ... then they wanted some food channel, but gives them very afraid to make changes and especially gives very scared vegetables.

When they come has never been the case of Crohn control. For example the last case I came because I wanted to lose weight, because they had to operate and before operating want to lose weight, but not control his chronic illness.

When they come to the office, I almost always tell that they have banned vegetables and that they have to keep trying and that they must know what they have to go removing. That's the message they convey me about their knowledge of food received by the professionals, then there are people who have collected information through internet and then go to pages ... But the message of health professionals is gradually going testing the foods they feel better and worse and eliminate them. I think it's a very deficient information, I think there is no one to sit with them and explain them food groups or foods usually sit but ... we agree that in all cases but no individual tolerance,tell you see you see removing and testing ... I do not think ... And grosso remove vegetables, give them a lot of fear. Because yes relate food with sprouts, maybe they have eaten something with lots of fiber as spinach or something like that and associated with an outbreak and no longer return to try more. So I think that nutritionists should be within public health and offer our knowledge, that nurse practitioners do not have and not have more specific knowledge and those do not. As in health centers, now is the nurse who carries chronic patient consultations and there's an important part of nutrition, they could also do group sessions with group of patients,share recipes ... that would help the social support network and create this network group within the different disease and there has to be a nutritionist to guide the various sessions. I'm not saying that maybe it has to be the same periocidad that nurses, for example in a cure, but if you must have some briefings, an evolution for the nutritional management consultant. Especially at the time it is diagnosed, because I think they are very lost at that time.

**What recommendations would you offer them?**

Because in general, I do tell them that we are gradually changing food go to other than a radical change, now they are quiet. And then we are gradually introducing elements. Quantified fruit and vegetables and depending on how they are telling me they gonna respond, which is generally good, because I modified.

**What is your perception of your experience?**

I think at first, is a disease that limits them a lot, especially in the social part, no ? Not being able to go out and not eat certain things, they do not know how you can sit, because they often come and abuse fried, which also feel very badly. Why there is much limitation at first, until they know themselves and know their disease, they are very limited in the social part ... which seems to be secondary, but the end is very important. So in this part they are very limited, because at home they feel as safer at home they control what they eat, even if they have to do a different meal for the rest of the family, but when they leave or when there are family celebrations, I think there are suffering, are very limited.

I also believe that there should be greater support and the healthcare team should also make references to web's reliable so that they can learn scientific evidence.

**Interview K**

**Nursing assistant**

**What do you think it impacts them most people when they tell them they have Crohn?**

Well I speak from my experience, I was lost, I was lost because it was a subject that is completely unaware, and then it made the grave error of internet ... and it was disastrous, for me that was frustrating because for me either I was so bad, as put there, but really something happened ... there was sometimes eating astringents things and I kept making the same number of depositions, though, medication and everything, and we think you are lost. But hey, once it exceeded a little ...

**Did you ask someone?**

Yes, I asked the doctors, especially doctors and a companion who has also, that girl said "the key is not grated" and is quite right, the truth is that yes, that's a pretty nasty disease, especially as it entails, after that, be aware of having a clean bathroom, especially ... it's a little asquerosita, but ...

**So you think what strikes you most is ignorance**

Of course, above all that, yes but hey, the rest then gradually you're feeling better, but at first you find yourself lost, because it was a subject that did not control ...

**Who do you think people are supported with this disease?**

Oops! Man primarily in the family, but of course, as is a disease in which you appear not really sick, you take a lot of medication but do not look anything and I know ... in the family, in couples. Always Procures ask the sick when you walk into the room ... the truth is that the cases I have known were usually always lonely, but I understand you know ?, if they were with active disease, the truth is that is a bit unpleasant you have the roomful not you understand me?

**Let family ...**

I've always wondered patients Hey how are you? And people Super -well, I've always told things ...

**What difficulties are think over the disease?**

Man in food, which is essential medication ... depends ... .. do not know. They are usually patients with treatment achievers ...

**Have you received or have you heard any limitations in your life?**

No, all food, you ask ... no, you know what happens to patients I have treated on the ground as they have been pretty bad, either came to ask or talk more. And the fact imagine a person 40 years admitted to hospital more than a month, because that already limits for everything, for your work visa for their personal life and for all, that is clear. The fundamental thing was that were restored as soon as possible ... But it is also true that they are patients who have long with the disease as either, we know it as that, let you take it as an outbreak and it will pass

**Mmm What do you think professionals rely more?**

It depends, really, is that now I have the image of a patient who was a bit dry and well ... because maybe in the auxiliary who are the more time they spend in quotes, note that they are the longest pass ... because good to us right now with the volume of patients we have, neither we can entertain as much as we would like, then of course the auxiliary between that make the bed, if the washing, cleaning that make them the room, entertain or what .. is therefore discharged more. There are assistants who really know how to listen, and also they are very marujas ...

**You've talked about a case that was like a little 'dry' do you think that most patients share common personality patterns?**

No, no, I think not, what happens is that man, for each disease each carries his or her things, as you know or can you know? I know, we're going to be all ...

**What professional do you think would be best suited to keep track?**

Psychologists believe that psychological support is essential because you will head, because of course when you start to bleed and such ... sometimes you can not leave home, then it's horrible, you think you need to be near a toilet continuously, it is maddening and at home do not really understand ... but psychologists at certain stages of the disease and in some cases come very well, because it really is it's a bit humiliating this disease ...

**Humiliating?**

Sure, the symptoms and because you always have to have a bathroom nearby ...

**You are offered the opportunity psychologist ...**

I think not, because they take from doctor to doctor, and also no hospital, no psychiatrist ... Well in social security itself that there are psychologists and other derived

**What coping strategies for coping perform?**

I guess, I do not know, the truth is it happens ...

**What do you think needs that are not covered from your vision plan?**

Ay ! Because people understand you, is not, is that you have to suffer it , I think, that you understand ... Understanding you know? It is that nobody you know? It seems that nothing happens, which is no more diarrhea, but it is not

**So you think if you are covered?**

No, for understanding, if the patient needs support is not given, we will question what is the disease that is the most rapid recovery possible, but actually remain without cover many aspects that are not psychological ... the family is involved if a young little boy, is the means or yes, but I think if it's an older person, for longer

**When would gain treatments, you talk to them about side effects?**

No, in my case because I've always treated people who already have the disease a long time, then you already know everything and do not ask ...
